# LncRNA SFTA1P mediates positive feedback regulation of the Hippo-YAP/TAZ signaling pathway in non-small cell lung cancer

**DOI:** 10.1038/s41420-021-00761-0

**Published:** 2021-11-29

**Authors:** Bowen Zhu, Megan Finch-Edmondson, Kim Whye Leong, Xiaoqian Zhang, Mitheera V., Quy Xiao Xuan Lin, Yaelim Lee, Wei Ting Ng, Huili Guo, Yue Wan, Marius Sudol, Ramanuj DasGupta

**Affiliations:** 1 Genome Institute of Singapore (GIS), Agency for Science, Technology and Research (A*STAR), Singapore, 138672 Singapore; 2 Department of Physiology, NUS Yong Loo Lin School of Medicine, National University of Singapore, Singapore, 117593 Singapore; 3 Mechanobiology Institute (MBI), National University of Singapore, Singapore, 117411 Singapore; 4 Cancer Science Institute of Singapore, National University of Singapore, Singapore, 117599 Singapore; 5 Institute of Molecular and Cell Biology (IMCB), Agency for Science, Technology and Research (A*STAR), Singapore, 138673 Singapore; 6 Present Address: Icahn School of Medicine at Mount Sinai, New York City, NY 10029 USA

**Keywords:** Cell signalling, Cancer genomics

## Abstract

Long non-coding RNAs (lncRNAs) regulate numerous biological processes involved in both development and carcinogenesis. Hippo-YAP/TAZ signaling, a critical pathway responsible for organ size control, is often dysregulated in a variety of cancers. However, the nature and function of YAP/TAZ-regulated lncRNAs during tumorigenesis remain largely unexplored. By profiling YAP/TAZ-regulated lncRNAs, we identified SFTA1P as a novel transcriptional target and a positive feedback regulator of YAP/TAZ signaling. Using non-small cell lung cancer (NSCLC) cell lines, we show that SFTA1P is transcriptionally activated by YAP/TAZ in a TEAD-dependent manner. Functionally, knockdown of SFTA1P in NSCLC cell lines inhibited proliferation, induced programmed cell death, and compromised their tumorigenic potential. Mechanistically, SFTA1P knockdown decreased TAZ protein abundance and consequently, the expression of YAP/TAZ transcriptional targets. We provide evidence that this phenomenon could potentially be mediated *via* its interaction with TAZ mRNA to regulate TAZ translation. Our results reveal SFTA1P as a positive feedback regulator of Hippo-YAP/TAZ signaling, which may serve as the molecular basis for lncRNA-based therapies against YAP/TAZ-driven cancers.

## Introduction

The Hippo-YAP/TAZ signaling pathway is an evolutionarily conserved signaling pathway involved in organ size control, embryonic development, and tumorigenesis [1, 2]. Activation of Hippo signaling leads to the phosphorylation of two Hippo pathway effectors, namely Yes-associated protein (YAP) and its paralog, WW domain-containing transcription regulator protein 1 (WWTR1 or TAZ). Phospho-YAP/TAZ are retained in the cytoplasm through their interactions with 14-3-3 proteins and are eventually degraded by ubiquitin-mediated proteolysis [3–5]. Inactivation of the Hippo pathway leads to the accumulation of unphosphorylated YAP and TAZ and their nuclear localization, where they activate target gene expression through their interactions with co-regulatory transcription factors, such as TEA domain-containing transcription factors (TEADs) [6, 7]. TEADs modulate gene expression by binding directly to promoters and/or enhancers of their target genes via consensus TEAD binding sites [6, 8]. Transcriptional targets of YAP and TAZ are involved in the regulation of a wide spectrum of cellular processes, including cell cycle, epithelial-mesenchymal transition (EMT), cell polarization, and stem cell pluripotency [9, 10]. For example, CYR61, a bona fide YAP/TAZ target gene, was found to enhance tumorigenesis [11], neo-angiogenesis [12], and metastasis [13]. Therefore, YAP/TAZ transcriptional targets exert crucial roles in oncogenic transformation [14, 15].

Non-coding RNAs, including microRNAs and long non-coding RNAs (lncRNAs), are transcripts that do not encode proteins, and their deregulation has been linked to cancers [16, 17]. Previous studies have uncovered the association between YAP/TAZ and microRNAs in cancer [18–23]. However, the role of lncRNAs that are transcriptionally regulated by YAP and TAZ remains poorly understood. Recently, a number of studies suggested that lncRNAs are involved in the YAP/TAZ-driven tumor progression. For instance, YAP/TAZ-driven transcriptional repression of the lncRNA, NORAD, was found to be responsible for metastasis of lung and breast cancer [24]. LncRNA, MAYA, has been reported to play a vital role in activating YAP through the methylation and consequent inhibition of MST1 during bone metastasis [25]. Altogether, these studies reveal critical functions of YAP/TAZ-associated lncRNAs in the regulation of signaling pathways that modulate a variety of cell-biological processes, further alluding to their diverse functions in cancer.

In this report, we present the identification and functional characterization of a novel lncRNA target of the Hippo-YAP/TAZ signaling. By analyzing RNA-seq data generated in cells overexpressing either YAP or TAZ, we identified SFTA1P, the expression of which is transcriptionally regulated by YAP/TAZ in a TEAD dependent manner. Functionally, we show that SFTA1P contributes to YAP/TAZ-mediated regulation of non-small cell lung cancer (NSCLC) cell proliferation and survival, therefore conferring tumorigenic properties. We also elucidate a positive feedback regulatory function of SFTA1P, whereby SFTA1P itself is required for the TAZ protein expression and YAP/TAZ/TEAD-driven gene transcription. These findings reveal new insights into the regulation and function of the lncRNAs that are transcriptionally regulated by YAP/TAZ and specifically highlight SFTA1P as a potential therapeutic target for the treatment of YAP/TAZ-associated cancers.

## Results

### SFTA1P is a transcriptional target of YAP/TAZ/TEAD

**Fig. 1:**
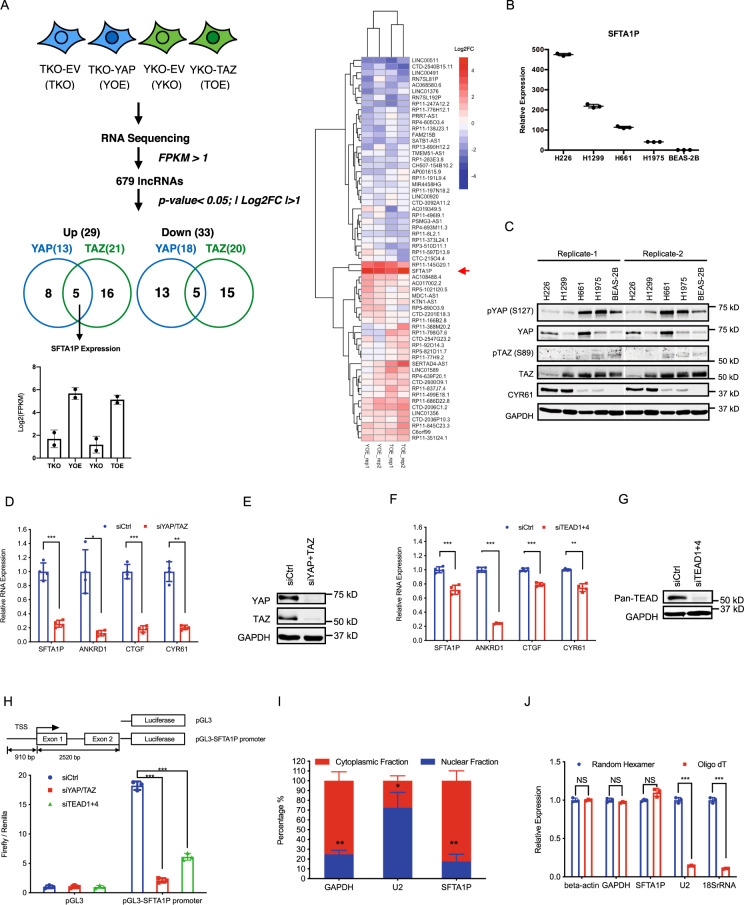
SFTA1P is transcriptionally regulated by YAP/TAZ/TEAD in NSCLC cell lines. **A** A schematic illustration of the lncRNA profiling in MKN28 gastric cancer cells. SFTA1P was identified as a candidate target of YAP/TAZ. Briefly, engineered YAP/TAZ-knockout cells (YKO/TKO) were transduced with empty vector (TKO-EV or TKO) or active form of YAP (TKO-YAP or YOE)/TAZ (YKO-TAZ or TOE). RNA sequencing (*n* = 2 biologically independent samples) was performed to identify the differentially expressed lncRNAs with the criteria indicated. Venn diagram shows the lncRNAs that were up/downregulated upon overexpression of YAP/TAZ. Bar chart plots the relative expression of SFTA1P using Fragments Per Kilobase of transcript per Million mapped reads (FPKM). The heatmap demonstrates the relative expression of differentially expressed lncRNAs upon YAP/TAZ overexpression. The LOG2 fold-change of each lncRNA upon YAP/TAZ overexpression in two replicates were calculated and plotted. The red arrow pinpoints SFTA1P. **B** The expression of SFTA1P in lung cell lines was evaluated by RT-qPCR. The relative expression of SFTA1P was calculated by normalizing the SFTA1P expression in the indicated cell lines against that in the BEAS-2B cell line. Mean ± SD, *n* = 3 (biological replicates). **C** Western blot analysis of pYAP (S127), YAP, pTAZ (S89), TAZ, CYR61, and GAPDH in the indicated lung cell lines. **D**–**G** Knockdown of YAP (**D**–**E**), TAZ (**D**–**E**), and TEADs (**F**–**G**) in H1299 cells. The knockdown efficiencies were evaluated by western blot analysis in (**E**, **G**). The RNA expression of SFTA1P, ANKRD1, CTGF, and CYR61 were measured by RT-qPCR following the knockdowns of YAP and TAZ (**D**) or TEAD1 and TEAD4 (F), Mean ± SD, *n* = 4 (biological replicates). **H** The transcriptional activity of the regulatory sequence in the vicinity of SFTA1P TSS was assessed by dual-luciferase assay upon co-transfection of the reporter plasmids and siRNAs against YAP, TAZ, or TEADs in H1299 cells. A schematic illustration of the promoter sequence cloned into a pGL3-Basic vector was shown in the top panel: the 910 bp upstream and 2520 bp downstream sequence of the SFTA1P’s TSS (overlapping with the 1st exon, 1st intron, and the partial sequence of the 2nd exon) was examined. Mean ± SD, *n* = 3 (biological replicates). **I** Subcellular localization of SFTA1P was determined by RT-qPCR following subcellular fractionation in H1299 cells. The expression of GAPDH (cytoplasmic marker), U2 snRNA (nuclear marker), and SFTA1P were each quantified and normalized to their corresponding expression levels in the whole-cell lysate. The percentage of the expression in cytoplasmic fraction or nuclear fraction versus the summation of them were calculated and plotted. Mean ± SD, *n* = 3 (biological replicates). **J** The presence of Poly(A) tail in SFTA1P transcript was measured by RT-qPCR following reverse transcription using either random hexamer or oligo-dT. The relative expression of each gene was obtained by normalizing against the expression of beta-actin. The comparisons were calculated by normalizing the relative expression in the cDNA library synthesized using random hexamer. Mean ± SD, *n* = 3 (biological replicates). Statistical significance was determined using Student’s *t*-test, *p < 0.05, **p < 0.01, ***p < 0.001.

To gain insights into the regulatory role of YAP/TAZ in controlling lncRNA gene expression, we re-analyzed the RNA sequencing data from our previously published study focusing on the identification of common and unique signatures of YAP and TAZ transcription in gastric cancer cells [26]. As previously described, YAP was overexpressed in CRISPR/Cas9-mediated TAZ knockout cells, while a converse overexpression of TAZ was conducted in YAP knockout cells (Fig. 1A). As proof-of-concept, reverse transcription-quantitative polymerase chain reaction (RT-qPCR) was performed to examine the expression of representative YAP/TAZ target genes (ANKRD1, CTGF, and CYR61) [1], which revealed marked upregulation upon YAP/TAZ overexpression (Fig. S1A-C). RNA-seq data and differential expression analysis [27, 28] yielded 679 lncRNAs (FPKM > 1), in which 62 lncRNAs were differentially expressed (Fig. 1A, Supplementary Table 1). Specifically, 29 lncRNAs were upregulated, and 33 were downregulated with an absolute value of Log2 fold-change (Log2 FC) >1 (Fig. 1A). Amongst the upregulated lncRNAs, 5 were increased in both YAP and TAZ overexpression, whereas the remaining lncRNAs responded to only specific overexpression of either YAP or TAZ (Fig. 1A). Similarly, overexpression of YAP/TAZ downregulated 33 lncRNAs, including 5 that were influenced by both YAP and TAZ. Given that YAP and TAZ primarily act as transcriptional co-activators [29–31], we focused our analysis on the upregulated lncRNAs, which were more likely to be direct transcriptional targets of YAP and TAZ. As illustrated in Figs. 1A and S1D, SFTA1P was the topmost upregulated lncRNA upon YAP/TAZ overexpression, which thus became the focus of our study.

Given that SFTA1P is highly expressed in the lung compared to other tissues [32] (Fig. S1E), we hypothesized that SFTA1P may represent a novel YAP/TAZ-regulated functional target in the lung. To determine the expression of SFTA1P in lung cell line models, we performed RT-qPCR in four NSCLC cell lines (H226, H1299, H661, and H1975) and an immortalized normal human bronchial epithelium cell line (BEAS-2B). SFTA1P was highly expressed in H226 and H1299 cell lines, with moderate expression in H661 and H1975, and negligible expression detected in BEAS-2B (Fig. 1B). Western blot analysis demonstrated that total YAP protein was more abundant in the H226 and H661 cell lines, whereas total TAZ protein was highly expressed in all the cell lines except for in H226 (Fig. 1C). However, the inactive phosphorylated forms of YAP and TAZ (pYAP and pTAZ) were negligibly expressed in H226 and H1299 cells, which was consistent with abundant expression of CYR61, suggesting active YAP/TAZ signaling in these two cell lines (Fig. 1C). In contrast, pYAP and pTAZ expression were more prominent in H661, H1975, and BEAS-2B cells, which was in accordance with moderate or low expression of SFTA1P and CYR61 (Fig. 1B, C). Furthermore, Spearman correlation analysis revealed that SFTA1P expression positively correlated with the expression of ANKRD1, CTGF, and CYR61 (*R* > 0.6), suggesting that SFTA1P expression is indeed associated with the activity of Hippo-YAP/TAZ signaling in lung cell lines (Fig. S1F–H).

To determine the role of YAP and TAZ in the regulation of SFTA1P expression, we silenced YAP and TAZ using siRNAs in NSCLC cell lines. RT-qPCR revealed that co-knockdown of YAP and TAZ led to a significant reduction of SFTA1P expression in H1299 (Fig. 1D, E), H661 (Fig. S1I, J), and H1975 (Fig. S1K, L) cell lines, along with a concomitant decrease in the expression of target genes ANKRD1, CTGF, and CYR61 (Figs. 1D, S1J, L). These observations are consistent with the notion that SFTA1P behaves like a bona fide YAP/TAZ target gene and that YAP/TAZ activity is required for SFTA1P expression in NSCLC cells. Next, we investigated whether YAP/TAZ-driven SFTA1P expression is TEAD-dependent. The expression of the four TEAD genes was examined by RT-qPCR [33, 34], which revealed that TEAD1 and TEAD4 were more abundant than TEAD2 and TEAD3 in H1299 cells (Fig. S1M). Subsequently, co-knockdown of TEAD1 and TEAD4 in H1299 cells significantly reduced the expression of SFTA1P as well as the three representative transcriptional targets of YAP/TAZ (Fig. 1F, G), implying that SFTA1P expression is indeed TEAD-dependent. To explore whether SFTA1P is directly regulated by YAP/TAZ/TEADs, we analyzed a previously published ChIP-Seq dataset [35] that scrutinized potential YAP/TAZ/TEAD binding sites in the vicinity of SFTA1P’s transcriptional start site (TSS). Interestingly, two YAP/TAZ/TEAD binding peaks were found in the region spanning from −2500 bp to +2500 bp of SFTA1P’s TSS (Fig. S1N) [35]. We further examined the sequence within the −910 to +2520 bp region flanking the TSS of SFTA1P, which unveiled seven consensus TEAD binding sequences (5′-C/AATTCC-3′ and 5′-GGAATT/G-3′) [6, 8] (Fig. S1O). To validate the functionality of these putative transcription factor-binding sites, we performed luciferase reporter assays using a reporter construct containing the sequence from −910 to +2520 bp neighboring the SFTA1P TSS at the 5’ end of a firefly luciferase gene (Fig. 1H). The recombinant plasmid exhibited a higher luciferase activity in comparison to the control plasmid that lacks the SFTA1P regulatory sequence, indicating that the region is required for SFTA1P expression (Fig. 1H). Interestingly, the knockdown of either YAP/TAZ or TEAD1/4 significantly diminished luciferase activity (Fig. 1H), suggesting the transcriptional regulation of SFTA1P is dependent on YAP/TAZ/TEAD. In order to gain more insights into this notion, we generated two mutants bearing deletions of the typical TEAD binding sequence, 5′-GGAATT-3′ (Fig. S1O). As reflected in Fig. S1P, SFTA1P-Promoter del1 (site 1264–1269) significantly compromised the promoter activity, whereas SFTA1P-Promoter del2 (site 1736–1741) appeared to be relatively dispensable. Taken together, our results suggest SFTA1P is a direct transcriptional target of the YAP/TAZ/TEAD complex.

LncRNA functionality is often influenced by their subcellular localization [36]. We, therefore, determined the subcellular localization of SFTA1P using cell fractionation and subsequent RT-qPCR in H1299 cells. Similar to GAPDH (cytoplasmic control), and in sharp contrast with the nuclear marker U2 spliceosomal snRNA [37], the majority of SFTA1P (>80%) was detected in the cytoplasmic fraction (Fig. 1I). As polyadenylation (poly(A)) is known to direct the translocation of transcripts to the cytoplasm [38], SFTA1P was assessed for the presence of a poly(A) tail by comparing its cDNA synthesized using either random hexamers or oligo-dT primers. Notably, similar to GAPDH and beta-actin, SFTA1P was equally amplified using both RT methods (Fig. 1J), indicating that SFTA1P is polyadenylated. In contrast, nuclear U2 snRNA and 18S rRNA that lack poly(A) tails were poorly detected when their cDNAs were synthesized using oligo-dT probes (Fig. 1J) compared to those using random hexamers. Altogether, these results revealed that SFTA1P is predominantly localized to, and therefore might exert its function in the cytoplasm.

### Loss of SFTA1P inhibits the growth and tumorigenic potential of NSCLC cells

**Fig. 2:**
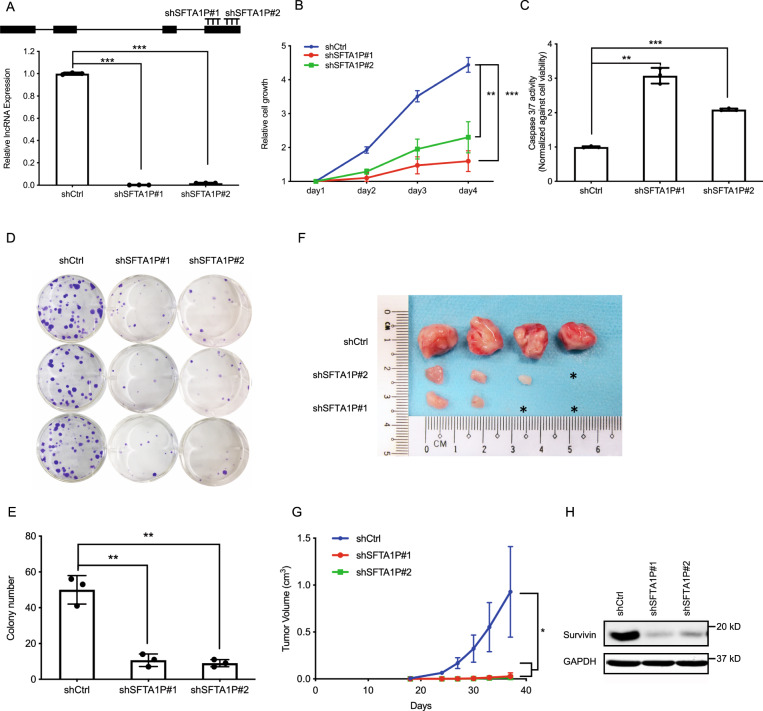
Loss of SFTA1P inhibits tumor growth in vitro and in vivo. **A** Schematic annotations of SFTA1P and two shRNAs against the 4th exon of SFTA1P (top panel). The knockdown efficiencies were evaluated by RT-qPCR in H1299 cells (bottom panel). **B** The cell growth curve was measured by cell viability assay following shRNA-mediated silencing of SFTA1P. The relative viabilities of each treatment on day 4 were subjected to statistical analysis. Mean ± SD, *n* = 3 (biological replicates). **C** Caspase 3/7 assay measures the caspases activity upon knockdown of SFTA1P in H1299 cells. The relative caspase 3/7 activities were obtained by normalizing the raw readouts against the corresponding cell viabilities estimated by CellTiter Glo. Mean ± SD, *n* = 3 (biological replicates). **D**–**E** Colony formation assay (**D**) and the corresponding quantification (**E**) in H1299 cell upon shRNA transduction. Mean ± SD, *n* = 3 (biological replicates). **F**–**G** A representative image (**F**) and quantitative analysis (**G**) of the volumes of tumors formed after subcutaneously transplanting 4 × 10^5^ H1299 cells transduced with shRNAs. Mean ± SD, *n* = 3 (biological replicates). **H** Western blot analysis of survivin expression upon the knockdown of SFTA1P. Statistical significance was determined using Student’s *t*-test, *p < 0.05, **p < 0.01, ***p < 0.001.

YAP and TAZ are potent oncogenes that promote tumor growth by regulating the balance between cell proliferation and apoptosis [10]. To determine whether SFTA1P could also contribute to these functions, we conducted loss-of-function studies in H1299, H661, and H1975 cell lines. Knockdown of SFTA1P using two individual shRNAs (shSFTA1P #1 and #2) resulted in a significant loss of its expression in NSCLC cells (Figs. 2A, S2A, B). Notably, in vitro growth curve assays revealed markedly reduced growth rate and likely proliferative capacity of cells upon SFTA1P-knockdown, compared to control in H1299 (Fig. 2B), H661 (Fig. S2C), and H1975 cells (Fig. S2D). Moreover, SFTA1P knockdown resulted in a significant upregulation of pro-apoptotic caspase-3/7 activity in H1299 (Fig. 2C), H661 (Fig. S2E), and H1975 (Fig. S2F) cells, indicating increased cell apoptosis in the absence of SFTA1P. Furthermore, loss of SFTA1P significantly inhibited colony formation in H1299 cells (Fig. 2D, E), suggesting that SFTA1P may regulate the tumorigenic potential of NSCLC cells in vitro. To gain more insights into the tumorigenic function of SFTA1P in vivo, shSFTA1P- and shCtrl-transduced H1299 cells were subcutaneously injected into immunocompromised mice and monitored for tumor growth. Compared to shCtrl-treated cells, which generated large tumors, shSFTA1P-treated cells resulted in either markedly reduced or no tumor formation (Fig. 2F, G). In line with the observations, knockdown of SFTA1P led to a concomitant reduction in the expression of survivin (Fig. 2H), a known inhibitor of apoptosis (IAP), and caspase-3/7 activity [39, 40], further corroborating the anti-apoptotic function of SFTA1P. Altogether, our in vitro and in vivo data suggest that SFTA1P may play a pro-proliferative and anti-apoptotic role in NSCLC tumorigenesis.

### SFTA1P regulates TAZ through a positive feedback loop

**Fig. 3:**
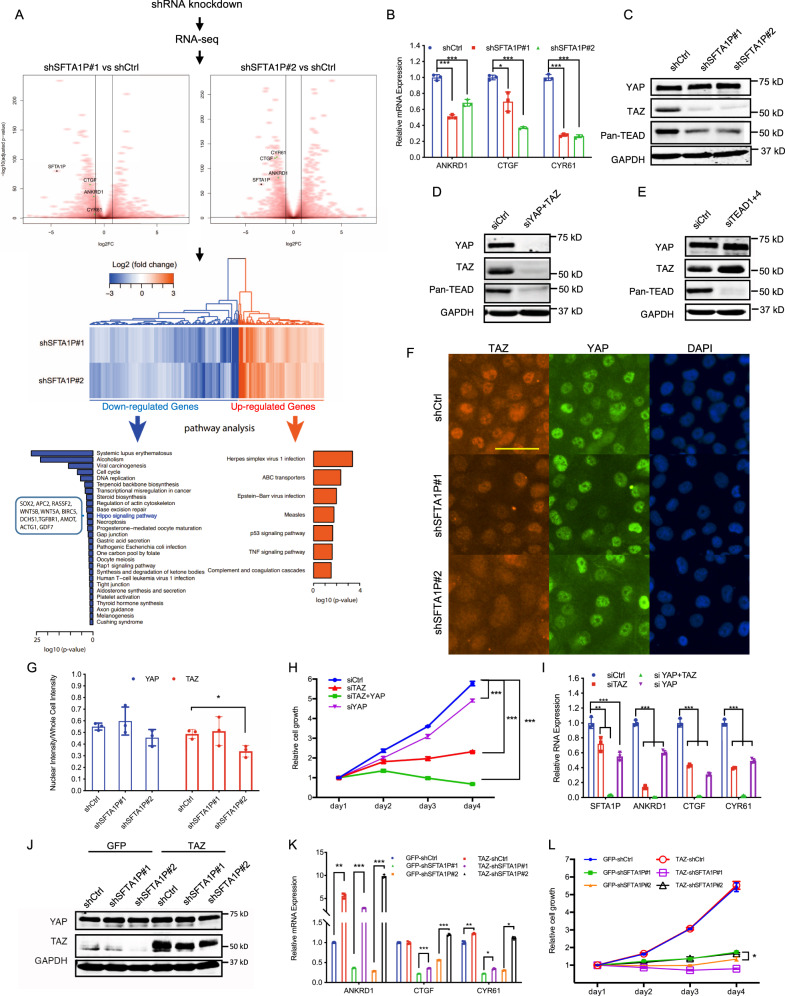
SFTA1P mediates a positive feedback regulation of the Hippo-YAP/TAZ signaling pathway by targeting TAZ. **A** RNA-seq analysis revealed the differentially expressed genes following the knockdown of SFTA1P in H1299 cells. Volcano plots show the LOG2 fold-changes in gene expression (*x*-axis) and the −Log10 of the Benjamini-Hochberg adjusted *P*-values (*y*-axis) between the cells treated with shSFTA1P#1 (left panel) or shSFTA1P#2 (right panel) versus shCtrl. *n* = 2 (biological replicates). SFTA1P and transcriptional targets of YAP/TAZ were highlighted in black and green, respectively. Heatmap shows deferentially expressed genes following the shRNA-mediated knockdown of SFTA1P. The up/down-regulated genes were subjected to GO analysis, in which the genes were significantly associated with the indicated pathways. **B** Three transcriptional targets of YAP/TAZ/TEADs, namely ANKRD1, CTGF, and CYR61, were evaluated by RT-qPCR in H1299 cells upon SFTA1P knockdown. Mean ± SD, *n* = 3 (biological replicates). **C** Western blot shows the protein levels of YAP, TAZ, and TEADs upon SFTA1P knockdown. **D**–**E** Western blot demonstrates the protein levels of YAP, TAZ, and TEADs upon the siRNA-mediated knockdowns of YAP + TAZ or TEAD1 + TEAD4. **F**–**G** Subcellular localization of YAP/TAZ was examined by immunofluorescent staining (**F**) and quantification (**G**) of YAP, TAZ, and nuclei (DAPI) in H1299 cells. Scale bar = 100 µm. Mean ± SD, *n* = 3 (biological replicates). **H** Cell growth curves of H1299 cells following the treatments of indicated siRNAs were measured by CellTiter Glo. Mean ± SD, *n* = 3 (biological replicates). The relative viabilities of each treatment on day 4 were subjected to statistical analysis. **I** RT-qPCR analysis of SFTA1P and YAP/TAZ transcriptional targets upon siRNA treatment in H1299. Mean ± SD, *n* = 3 (biological replicates). **J** Western blot shows protein levels of YAP and TAZ following the transduction of GFP, TAZ, and shRNAs in the H1299 cells. **K** RT-qPCR analysis of YAP/TAZ transcriptional targets following the transduction of GFP, TAZ, and shRNAs in the H1299 cells. **L** Cell growth curves were measured by cell viability assay following the transduction of GFP, TAZ, and shRNAs in the H1299 cells. Mean ± SD, *n* = 3 (biological replicates). The relative viabilities of each treatment on day 4 were subjected to statistical analysis. All statistical analysis was conducted using Student’s *t*-test, *p < 0.05, **p < 0.01, ***p < 0.001.

To gain better insights into the molecular function and downstream targets of SFTA1P, we performed RNA-seq in H1299 cells following the knockdown of SFTA1P. Compared to shCtrl-treated cells, 516 genes, including ANKRD1, CTGF, and CYR61, were significantly downregulated, while 296 genes were upregulated following the knockdown of SFTA1P (Fig. 3A, Supplementary Table 2). Consistently, KEGG pathway analysis of differentially expressed genes revealed that the genes downregulated upon SFTA1P knockdown were significantly enriched for the Hippo signaling pathway (Fig. 3A). Other enriched pathways included those involved in the regulation of actin cytoskeleton, gap and tight junctions, and cell cycle/DNA replication, thereby drawing parallels between SFTA1P and the known functions of Hippo signaling in cell growth, polarity, and cytoskeletal regulation (Fig. 3A) [1, 41–45]. We, therefore, speculated that SFTA1P could serve as a positive feedback regulator of YAP and TAZ to potentiate YAP/TAZ signaling during tumorigenesis. To interrogate this hypothesis, we first examined the expression of YAP/TAZ transcriptional targets (ANKRD1, CTGF, and CYR61) by RT-qPCR. Remarkably, shSFTA1P significantly reduced the expression of all three genes in H1299 (Fig. 3B) and H661 (Fig. S3A) cells, as well as that of CYR61 in H1975 cells (Fig. S3B). We then investigated the expression of the primary regulators that modulate the expression of these target genes, namely, YAP, TAZ, and TEADs. Intriguingly, the protein expression of TAZ and TEADs, but not YAP, were markedly reduced upon SFTA1P knockdown in H1299 cells (Figs. 3C, S3C). To determine the order of regulation of TAZ and TEADs, we performed siRNA knockdown of YAP/TAZ and TEADs, respectively. Depleting YAP and TAZ caused a reduction in TEADs but not vice versa (Fig. 3D, E), suggesting that the changes in TEAD protein expression might be the result of compromised YAP/TAZ activity. Consistently, knockdown of SFTA1P also reduced the expression of TAZ and CYR61 in protein levels in H661 (Fig. S3D) and H1975 cells (Fig. S3E). In order to determine whether SFTA1P can influence the subcellular distribution of YAP and TAZ, a surrogate marker of their transcriptional activity, we employed immunofluorescence in cells transduced with shRNAs targeting SFTA1P. As evident in Fig. 3F, G, while TAZ protein expression was subtly decreased in the nucleus, nuclear YAP expression remained unchanged in cells lacking SFTA1P. Consistent with the western blot data (Fig. 3C), quantification of fluorescence intensity also revealed a specific attenuation in TAZ protein level but not that of YAP (Fig. S3F). These data support the notion that TAZ may serve as a functional target of SFTA1P.

YAP and TAZ are believed, at least in part, to be functionally redundant [9]. However, TAZ is frequently upregulated in NSCLC cell lines [46] and was also found to be frequently amplified in NSCLC clinical specimens [47] (Fig. S3G). To determine whether the loss of TAZ is sufficient to phenocopy the changes observed upon SFTA1P knockdown, YAP and TAZ were either individually or simultaneously silenced in H1299 cells (Fig. S3H). As expected, loss of both YAP and TAZ resulted in a marked reduction in cell proliferation. Interestingly, individual TAZ knockdown mirrored the effect induced by double YAP/TAZ knockdown, whereas individual YAP knockdown failed to do so (Fig. 3H). RT-qPCR analysis revealed that TAZ knockdown resulted in a slightly greater reduction of ANKRD1 and CYR61 than the loss of YAP (Fig. 3I), suggesting perhaps a more dominant function of TAZ in differentially regulating target genes involved in cell proliferation. To further test this notion, we overexpressed TAZ in STFA1P-knockdown H1299 cells. Indeed, TAZ overexpression could rescue the expression of YAP/TAZ target genes, albeit to different extent (Fig. 3J, K). However, the assessment of cell proliferation phenotypes revealed that TAZ overexpression, especially in shSFTA1P#2 knockdown cells, could only partially rescue the cell growth rate (Fig. 3L), indicating that TAZ overexpression may not be sufficient to fully rescue the plethora of changes caused by the knockdown of SFTA1P. Based on these data, we reason that the molecular function of SFTA1P may not be exclusively dependent on its ability to regulate TAZ expression. In summary, we conclude that SFTA1P can function as a positive feedback regulator of YAP/TAZ signaling by influencing, at least in part, the abundance of TAZ and its resultant transcriptional activity.

### SFTA1P regulates TAZ translation by interacting with its mRNA

**Fig. 4:**
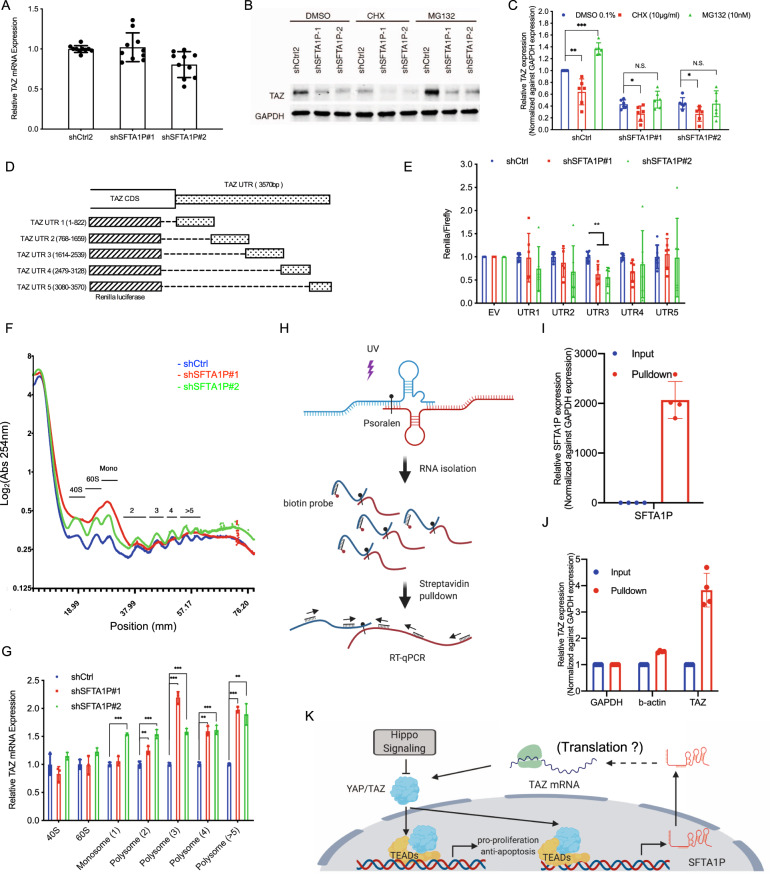
The 3′-UTR of TAZ mRNA is responsible for SFTA1P-mediated regulation. **A** RT-qPCR analysis of the TAZ mRNA upon shRNA treatments in the H1299 cells. Mean ± SD, *n* = 10 (biological replicates). **B**–**C** Western blot (**B**) and quantitative analysis (**C**) of TAZ protein expression in the H1299 cells stably transduced with shCtrl or shSFTA1Ps followed by the treatment of 1% DMSO, 10 µg/ml cycloheximide (CHX), and 10 µM MG-132 for 6 h. YAP, TAZ, and GAPDH were examined. Mean ± SD, *n* = 6 (biological replicates). **D** A schematic illustration of the 3′-UTR region of TAZ and the fragments subcloned into a psicheck2 reporter plasmid. **E** Dual-luciferase assay was conducted by transiently transfecting psicheck2 reporters into the shRNA-transduced. The readouts of renilla luciferase were normalized against the corresponding readouts of firefly luciferase. Mean ± SD, *n* = 6 (biological replicates). **F**–**G** Polysomal profiling followed by RT-qPCR. The cytosolic extracts obtained from shRNA-transduced cells were subjected to sucrose gradient centrifugation. The concentrations of ribosomes in each fraction are continuously estimated by UV absorbance (A254) (**F**). The TAZ mRNA associated with ribosomal subunits (40S and 60S), monosomes (80S), and polysomes are isolated and subjected to RT-qPCR analysis (**G**). Mean ± SD, *n* = 3 (technical replicates). The results was consistent in two independent experiments. **H** A schematic presentation of workflow. Briefly, double-stranded RNAs were crosslinked using Psoralen-UV method. A set of biotin-oligos against SFTA1P transcript was employed to enrich SFTA1P and the RNAs associated with it. The enriched RNA was then quantified using RT-qPCR. **I**–**J** RT-qPCR analysis of SFTA1P (**I**), GAPDH (**J**), b-actin (**J**), and TAZ (**J**) in the RNAs enriched by biotin-oligos against SFTA1P. Mean ± SD, *n* = 4 (biological replicates). **K** A proposed mechanism where SFTA1P, a YAP/TAZ transcriptional target, mediates a positive feedback regulation of the Hippo-YAP/TAZ signaling pathway by regulating TAZ at the level of translation. Statistical analysis was conducted using Student’s *t*-test, *p < 0.05, **p < 0.01, ***p < 0.001.

To determine the mechanism by which SFTA1P regulates TAZ protein expression, we first examined the expression of TAZ mRNA. RT-qPCR analysis revealed a marginal change in TAZ mRNA upon SFTA1P knockdown in H1299 cells (Fig. 4A), indicating a low possibility of regulation at the level of transcription or mRNA stability. Next, we interrogated whether SFTA1P regulates TAZ protein translation or stability. Global inhibition of protein translation by cycloheximide (CHX) led to a comparable loss of TAZ in the cells transduced with either control shRNA or shSFTA1P (Fig. 4B, C). In contrast, inhibiting proteasome-mediated protein degradation using MG132 increased TAZ protein levels (Fig. 4B, C). Importantly, MG132 treatment led to an accumulation of TAZ only in the shCtrl-transduced cells but not in the cells transduced with shSFTA1P (Fig. 4B, C), suggesting that SFTA1P might regulate TAZ expression upstream of its degradation. Altogether, the results indicate that the SFTA1P-driven TAZ regulation is likely at the level of protein translation. This hypothesis is also supported by the fact that SFTA1P primarily localizes to the cytoplasm (Fig. 1I), its likely site of activity, and the compartment where protein translation also occurs.

To further delineate the underlying mechanism of TAZ regulation by SFTA1P, we adopted three independent approaches. First, to determine whether translational regulation of TAZ by SFTA1P is dependent on its 3’-UTR, a luciferase reporter assay was utilized to pinpoint the specific region/s responsible for SFTA1P-mediated TAZ regulation. Out of five 3’-UTR fragments (UTR1–5) that were cloned into the reporter plasmid, only UTR-3 exhibited a consistent reduction of reporter activity upon SFTA1P knockdown (Fig. 4D, E), indicating a 3’-UTR-dependent regulation of TAZ translation. Second, we employed polysome-profiling to investigate the occupancy of ribosomes on TAZ mRNA (Fig. 4F, G). Briefly, sucrose gradient centrifugation was performed on the cytoplasmic extracts. The fractions containing various components or numbers of ribosomes, namely ribosomal subunits (40S and 60S), monosomes (80S), and polysomes, were isolated and subjected to RT-qPCR analysis to examine the levels of TAZ mRNA (Fig. 4F). Upon SFTA1P knockdown, RT-qPCR revealed a distinct accumulation of TAZ mRNA in the polysome fractions (Fig. 4G), which is shown to be associated with altered or even paused translation [48, 49]. Therefore, our data suggest a role of SFTA1P in the translational regulation of TAZ. Third, we asked whether the aforementioned regulation involves a direct interaction between SFTA1P and TAZ mRNA. Psoralen is commonly utilized to crosslink double-stranded hybrid-RNA regions amongst interacting RNA molecules under ultraviolet (UV) irradiation [50–53] (Fig. 4H). Thus, we applied Psoralen-UV crosslinking followed by RNA pulldown using biotin probes against SFTA1P in H1299 cells. The RNAs enriched after pulldown were then subjected to RT-qPCR analysis. As shown in Fig. 4I, SFTA1P was markedly enriched in the pulldown compared to the input sample. Strikingly, the pulldown fraction also revealed a four-fold enrichment of TAZ mRNA, but not that of the housekeeping gene, beta-actin. These observations demonstrate a direct interaction between the SFTA1P and TAZ mRNAs (Fig. 4J). Altogether, results from our studies suggest that SFTA1P mediates positive feedback regulation of the Hippo-YAP/TAZ signaling pathway by promoting the translation of TAZ mRNA, which is likely mediated by a direct interaction with the 3′-UTR of TAZ mRNA (Fig. 4K).

## Discussion

Our study identifies SFTA1P as a novel lncRNA that is transcriptionally regulated by YAP/TAZ in a TEAD dependent manner. This finding is consistent with previous observations that overexpression of YAP led to remarkable upregulation of SFTA1P in cell line models [7, 54]. While these early high-throughput studies have suggested a putative regulatory role of YAP in SFTA1P expression, our study provides the first direct evidence and mechanistic basis for SFTA1P regulation by YAP, TAZ, and TEADs. A previous transcriptional study suggested that around 20% of YAP/TAZ/TEADs target genes harbor YAP/TAZ/TEADs binding sites within 10 kb region of the respective TSS [35]. In agreement with it, we demonstrate that the sequence spanning from −910 to +2520 bp neighboring the SFTA1P’s TSS is responsible for its expression. The region comprises seven consensus TEAD binding sites, and at least one of them displayed YAP/TAZ/TEAD-dependent promoter activity in our cell line model. Therefore, SFTA1P is a novel transcriptional target of YAP/TAZ/TEAD.

Our data also reveal the tumorigenic function of SFTA1P in NSCLC cell lines. This is consistent with the known roles of YAP and TAZ, suggesting that SFTA1P is a functional target of YAP/TAZ. Interestingly, SFTA1P expression is relatively higher in the NSCLC cell lines compared to non-cancer lines, corroborating its tumorigenic potential in NSCLCs. Moreover, our functional data underscore the value of SFTA1P as a candidate therapeutic target in NSCLC, given that the depletion of SFTA1P resulted in the activation of pro-apoptotic pathways and remarkably compromised tumor growth/induction in vitro and in vivo. In addition, the lung-specific expression of SFTA1P indicates that targeting it therapeutically may mitigate adverse side-effects or toxicity in other tissues.

Mechanistically, we uncover a positive feedback regulation between SFTA1P and the Hippo-YAP/TAZ signaling pathway. Gene Ontology analysis indicates that Hippo signaling, as well as several pathways functionally relevant to Hippo signaling, were influenced upon SFTA1P knockdown, including cell cycle [1, 41], regulation of actin cytoskeleton [42, 43], Rap1 signaling pathway [55], and formation of tight junctions [44, 45]. Previous studies have identified NF2 and LATS2 as two transcriptional targets that also function as negative feedback regulators of Hippo-YAP/TAZ signaling [56]. Shen et al. reported a miR-130a-dependent positive feedback regulation of Hippo-YAP/TAZ signaling to be essential in organ size control and tumorigenesis [21]. Altogether, our study adds a novel layer of evidence to the regulatory potential of non-coding RNAs in the feedback regulation of Hippo-YAP/TAZ pathway activity.

We identify TAZ as a functional target of SFTA1P, whereby SFTA1P acts as a positive regulator that is required for YAP/TAZ signaling during NSCLC tumorigenesis. Findings from our study are consistent with recent reports that demonstrate a crucial role for TAZ in cancers. For example, the fusion of the TAZ gene with the gene that encodes CAMTA1 transcription factor is responsible for the majority of cases documenting a rare tumor known as Epithelioid Hemangio-Endothelioma (EHE) that primarily affects soft tissues, including lung [57, 58]. Altogether, our findings highlight the important function of SFTA1P in a TAZ-dependent regulation of Hippo-YAP/TAZ signaling and NSCLC tumorigenesis, which warrants further study.

Finally, our data shed light on the molecular basis underlying the regulatory function of SFTA1P in the control of TAZ expression by regulating its translation. In agreement with this notion, Strnadel et al. reported that YAP and TAZ are translationally regulated by the KRAS-eIF5A-PEAK axis in pancreatic cancers [59]. The 3′-UTR of mRNAs is believed to determine RNA stability, translation, and localization [60]. For instance, RNA “circularization” mediated by 3’-UTR of mRNAs and their interactors plays a pivotal role in translational control [61]. Concordant with these studies, we identify a specific region of TAZ 3′-UTR (+1614~+2539) to be responsible for SFTA1P-mediated regulation of TAZ expression. Furthermore, depleting SFTA1P resulted in the enrichment of TAZ mRNA in polysome fractions. We, therefore, speculate that the loss of SFTA1P may result in polysome pausing on the TAZ mRNA, thus leading to deficient translation. Further investigation will be critical to elucidate the specific mechanisms by which the interaction of SFTA1P with TAZ 3’-UTR affects polysome stalling and TAZ translation, as well as to explore other possible mechanisms such as those through competitive miRNA sponges [62, 63] or RNA binding proteins [64].

In summary, our study delineates a YAP/TAZ/TEAD-SFTA1P feedback loop that exerts an oncogenic role in NSCLC tumorigenesis. The findings provide basis for future research into the interplay between lncRNAs and Hippo-YAP/TAZ signaling, which may not only provide further understanding of ncRNA-mediated regulation of oncogenic signaling pathways, but also reveal their therapeutic potential in the clinic.

## Materials and methods

### Plasmid construction

For shRNA constructs, the scramble sequence (shCtrl) was described previously [65] and the two sequences targeting SFTA1P (shSFTA1P#1 and shSFTA1P#2), were designed using The Genetic Perturbation Platform (Broad Institute). The pLKO.1-puro (#8453; Addgene, Watertown, MA, USA) plasmid was used as the vector for shRNA expression. For the plasmids used in promoter analysis, the promoter sequence of SFTA1P was amplified from the genomic DNA of HEK293T with the restriction sites *Mlu I* and *BgI II* and cloned into the pGL3-Basic (E1751; Promega, Madison, WI, USA) plasmid. NEBaseChanger was utilized to design the primers used for generating deletions of TEAD-binding sites. For the UTR analysis, the fragments of TAZ’s 3′-UTR were each amplified from the cDNA library generated from the RNA of H1299 cells with the restriction sites *Not I* and *Sal I*, and subcloned into the 3’ to the Renilla gene within the modified psicheck-2 reporter plasmid (C8021; Promega), in which the original sequence between *Pvu I* and *Not I* sites (CGATCGCTCGAGCCCGGGAATTCGTTTAAACCTAGAGCGGCCGC) were replaced by the sequence (CGATCGCTCGACGAGCTCGCGGCCGCTCTAGAGTCGACGAATTCGATATCCTCGAGACTAGTCGGCCGC). For the FLAG-TAZ construct, the coding sequence of TAZ was amplified from the H1299 cDNA library with the restriction sites, *BamH I* and *Xho I*, and subcloned into the pLenti-CMV-GFP-puro (Addgene) plasmid digested by restriction enzymes, *BamH I* and *Sal I*. Oligo information can be found in Supplementary Table 3.

### Cell culture

Human gastric adenocarcinoma cell line MKN28, NSCLC lines H1299, H226, H661, H1975, and immortalized bronchial epithelium cell line BEAS-2B were cultured in RPMI-1640 (22400071; Gibco/Life Technologies, CarIsbad, CA, USA) supplemented with 10% fetal bovine serum (FBS) (10270106; Gibco/Life Technologies) and 100 U/mL Penicillin-Streptomycin (15140122 Gibco/Life Technologies). HEK293T cell line was cultured in high glucose DMEM supplemented with 1 mM sodium pyruvate, 10% FBS, and 100 U/mL penicillin-streptomycin. All cells were originally purchased from ATCC (Manassas, VA, USA), cultured under 5% CO_2_ at 37 °C, and tested negative for mycoplasma.

### Lentiviral packaging and viral transduction

The lentiviral plasmids (1.6 µg) were co-transfected with three packaging plasmids (gifts from Didier Trono), pMDLg/pRRE (0.8 µg), pMD2.G (0.4 µg) and pRSV-Rev (0.4 µg), into HEKH239T cells (1 × 10^6^) plated in 6 cm dishes using Lipofectamine 3000 transfection reagent (L3000150, Invitrogen, Waltham, MA, USA) according to the manufacturer’s instructions. The media was replaced with 3 mL of fresh media 24 h post-transfection. The viral supernatant was collected and filtered through a 0.45-µm filter 72 h after transfection before 200–400 µL of the filtered supernatant was used to infect cells in 12-well plates 24 h post-seeding. Positively transduced cells were selected with 1.5 µg/mL of puromycin (Sigma-Aldrich, St. Louis, MO, USA) for two days or longer till the mock transduced cells were dead.

### Western blot analysis

Cells cultured in six-well plates were washed with phosphate-buffered saline (PBS) before being lysed in RIPA buffer (89901; Thermo Scientific; Waltham, Massachusetts, United States.) supplemented with 1x Protease inhibitor (Roche cat. 11697498001) and 1x Phospho-STOP (Roche cat. 04906845001). Total protein was separated by SDS-polyacrylamide gel electrophoresis and then transferred onto a polyvinylidene difluoride membrane using a wet transfer method (constant current for 2 h). The membrane was blocked with 5% BSA in TBST (Tris Buffered Saline supplemented with 0.1% Tween 20) TBST for 1 h at room temperature before being incubated with primary antibody at 4 °C overnight. Antibodies: YAP (1: 500; sc-101199; Santa Cruz, Dallas, Texas, United States), TAZ (1:1000; #4883, Cell Signaling Technology, Danvers, MA, USA), YAP/TAZ (1:1000; #8418, Cell Signaling Technology), pan-TEAD (1:2000; #13295, Cell Signaling Technology), CYR61 (1:2000, #14479, Cell Signaling Technology), GAPDH (1:2000; sc25778, Santa Cruz, Dallas, Texas, USA). The membranes were then incubated with IRDye 680RD (1:5000) or IRDye 800CW (1:5000) (LI-COR, Lincoln, NE, USA) secondary antibodies at room temperature for 1 h before detection with the LICOR ODYSSEY scanner.

### Immunostaining and fluorescence microscopy

H1299 cells were plated onto ibidi slides (80806; Germany) two days prior to fixation. Cells were washed with cold PBS followed by fixation with cold 3.7% formaldehyde diluted in PBS. For staining, cells were washed and permeabilized with PBS supplemented with 0.2% Triton-X100 three times. The fixed cells were blocked using PBST (PBS + 0.1% Tween 20) supplemented with 1% BSA, then incubated with YAP (1:200; #14074; Cell signaling) and TAZ (1:200; 560235 BD Biosciences, San Jose, CA, USA) primary antibodies at 4 °C overnight. To detect YAP, TAZ, and nuclei, cells were incubated with secondary antibodies (1:1000; Alexa 488/594 from Life Technologies) and DAPI (1 drop in 500 µL; NucBlue, R37606, Invitrogen, Waltham, MA, USA) at room temperature for two hours. After three washes with PBST, 200 µL PBS was added and the slides were observed under a Nikon-Ti microscope.

### siRNA transfection

H1299 cells (7.5 × 10^4^), H661 cells (1.0 × 10^5^), and H1975 cells (8.0 × 10^4^) were seeded into a 12-well plate. After 24 h, cells were transfected using Lipofectamine 3000 transfection reagent (L3000150, Invitrogen) according to the manufacturer’s instructions. Cells were trypsinized 24 h post-transfection before being used for downstream assays, such as RNA isolation, protein extraction, and cell growth curve measurement. The siRNAs used in this study can be found in Supplementary Table 4.

### Cell proliferation assays

ShRNA- or siRNA-treated H1299 (2500), H661 (3000), and H1975 (3000) were plated into 96-well plates in 100 µL of fresh media on day 0, followed by being monitored every 24 h for 4 consecutive days. The cell viability was evaluated using CellTiter Glo reagent (G7572, Promega) according to the manufacturer’s instructions. Luminescence was measured using Tecan microplate reader (The Infinite M1000). The relative proliferation index on day 1 was normalized as 1.

### Cell apoptosis assays

ShRNA-treated H1299 (2500), H661 (3000), and H1975 (3000) were plated into 96-well plates in 100 µL of fresh media on day 0. 48 h after seeding, absolute caspase3/7 activity and the corresponding cell viability was evaluated by Tecan microplate reader after adding caspase-Glo 3/7 assay (G8092, Promega) and CellTiter Glo reagents, respectively according to the manufacturer’s instructions. The relative cell apoptosis was estimated by the ratio of the caspase-Glo 3/7 and CellTiter Glo readings.

### Colony formation assay

ShRNA-treated H1299 cells were seeded in a six-well plate with 500 cells per well. Fresh media was replenished every 4 days. The cells were incubated for 10 days followed by staining using crystal violet as described previously [66].

### RNA isolation and RT-qPCR

Total RNA was isolated from adherent cell lines using the RNeasy Mini Kit (Qiagen, cat. 74106) following the manufacturer’s recommendations before being evaluated using a NanoDrop. The total RNA (2 µg) was reverse transcribed using the SuperScript VILO cDNA Synthesis Kit (11754050, Invitrogen) according to the manufacturer’s instructions. The resultant cDNA was diluted by a factor of five before an aliquot was used for qPCR. qPCR was performed on the ABI-7300 instrument (Thermo Fisher Scientific) using the KAPA SYBR FAST qPCR kit (KK4601, KAPA, Sigma-Aldrich) and the gene-specific primers in a final volume of 10 µL. Relative gene expression was measured using the 2^−ΔΔCT^ method and normalized against 18S rRNA. The primers used for RT-qPCR can be found in Supplementary Table 5.

### Cell fractionation for RNA localization analysis

Cell fractionation was done using the PARIS kit (Waltham, MA, USA cat. AM1921) with QIAzol lysis reagent (QIAGEN cat. 79306). Cells cultured in a 35 mm dish were trypsinized and washed once with PBS before being resuspended in 1 mL of PBS. An aliquot of 200 µL (set aside as a ‘Whole-Cell Lysate’) was centrifuged at 400 × *g* for 5 min at 4 °C, resuspended in 200 µL disruption buffer, and kept on ice till the RNA/protein isolation. The remaining 800 µL was centrifuged at 400 × *g* for 5 min at 4 °C, after which the pellet was resuspended in 450 µL fractionation buffer, incubated for 10 min on ice, and then centrifuged to separate the cytoplasmic (supernatant) and the nuclear (pellet) fractions. The nuclear fraction was finally lysed in 250 µL disruption buffer and processed according to the manufacturer’s instructions. To isolate protein, all fractions were sonicated and centrifuged, followed by the collection of supernatant. To isolate RNA, four volumes of QIAzol were added to each fraction followed by adding chloroform (at a ratio of 0.2 mL chloroform per 1 mL QIAzol), mixed by vigorous shaking for 15 s followed by incubation at room temperature for 5 min. The mixture was then centrifuged at 12,000 × *g* for 15 min at 4 °C, after which the aqueous phase was collected and mixed thoroughly with an equal volume of pure ethanol to precipitate RNA. Precipitated RNA was subsequently isolated using Qiagen RNeasy mini column (Qiagen, cat. 74106) following the manufacturer’s instructions.

### Polyadenylation analysis of transcripts

The total RNA (1 µg) isolated from cultured cells were reversely transcribed using the SuperScript III First-Strand Synthesis System (18080051, Invitrogen) with different primers, namely oligo dT and random Hexamer, following the manufacturer’s instructions. The synthesized cDNA was then diluted by a factor of five and subjected to the qPCR analysis as described above.

### Tumor xenograft model

NSG (NOD.Cg-*Prkdcscid Il2rgtm1Wjl/*SzJ) male mice (InVivos/Jackson Laboratory, Singapore, stock no. 005557) were purchased at 6 weeks of age. H1299 cells (4.0 × 10^5^) stably transduced with shSFTA1P or scramble control (shCtrl) were resuspended in 20% Matrigel (BD Biosciences) diluted in saline and subcutaneously injected into a single flank of the randomly chosen NSG mice (*n* = 4/group). Tumor volume was measured every three days and estimated using the modified ellipsoidal formula [67, 68]:

$${\rm{Tumor}}\;{\rm{volume}}\left( {{\rm{mm}}^3} \right) = \frac{1}{2}({\rm{length}} \times {\rm{width}}^2)$$

Mice were euthanized, and the tumors were removed five weeks post-injection, before being assessed for size. Protocols for animal experiments were approved by the A*STAR Biological Resource Centre (BRC) Institutional Animal Care and Use Committee (IACUC) under protocol #151065.

### Luciferase assays

A number of 5000 H1299 cells were seeded in each well of a 96-well plate 1 day prior to transfection. For promoter analysis, cells were transfected with 50 ng mixture containing pGL3-Basic/pGL3-SFTA1P-promoter/pGL3-SFTA1P-promoter-del, 1 ng pCMV-Renilla luciferase vectors, and 10 nM siRNA. After 24-hour incubation, 50 µL media was replaced with the same volume of fresh media. For 3’-UTR analysis, transduced cells were seeded and subsequently transfected with 10 ng psicheck reporter. All reporter assays were performed at 48 h post-transfection using the Dual-Glo assay kit (E2930; Promega) according to the manufacturer’s instructions.

### RNA sequencing analysis in H1299 cell line

Total RNA was isolated using the miRNeasy kit (Qiagen, Chatsworth, CA, USA) according to the manufacturer’s instructions. The libraries were prepared using the TruSeq Stranded Total RNA Library Prep workflow with the Ribo-Zero Human/Mouse/Rat rRNA removal kit (Illumina) and sequenced using the HISEQ 4k paired-end method. Raw reads in libraries were trimmed along with adaptor removal using Trim Galore coupled with cutadapt [69]. Subsequently, trimmed reads of high base quality were aligned to human reference genome hg38 using STAR (-outFilterMultimapNmax 1, -outFilterMatchNminOverLread 0.80) [70]. Raw read counts in each gene were measured using featureCounts [71], followed by the calling of differentially expressed genes (DEG) using DESeq2 [72]. DEGs were determined with the absolute value of log2 fold change more than 0.8 and adjusted p-value <0.05. Further, modEnrichr was employed to analyze the pathways associated with the DEGs [73].

### Polysome analysis

H1299 cells were transduced with shCtrl or shSFTA1P. After 72 h, cells were incubated with CHX (100 mg/mL, Sigma-Aldrich) for 10 min and washed with PBS supplemented with 100 mg/mL CHX, followed by isolation of the cytoplasmic lysates in lysis buffer [10 mM Tris-HCl (pH 7.4), 5 mM MgCl_2_, 100 mM KCl, 2 mM DTT, 100 U/ml, SUPERase-In 100 U/ml, 100 μg/ml CHX (C4859, Sigma-Aldrich), Protease inhibitor (1XcOmplete, EDTA-free, Sigma-Aldrich). The crude lysates were then fractionated by ultracentrifugation through 15–50% linear sucrose gradients. In total, 15 fractions were collected and subjected to Phenol–Chloroform extraction followed by glycogen precipitation. RNA extracted from each fraction was analyzed by RT-qPCR.

### RNA pull-down assay

H1299 cells were pre-incubated with 0.44 mg/ml Psoralen-TEG-Azide (1352815–11–2; BERRY&ASSOCIATES, Dexter, MI, USA) in prior to UV irradiation (365 nm). RNA was then isolated using Trizol (15596018, Life Technologies). Three aliquots of RNA (40 µg each) in 300 µL nuclease-free water were individually hybridized with 4 µL pooled antisense biotin-labeled DNA oligomers (100 µM) against SFTA1P in 510 µl RNA hybridization buffer (750 mM NaCl, 1% SDS, 50 mM Tris-Cl pH 7.0, 1 mM EDTA) supplemented with 90 µL formamide (10%) and 2 µL Superase-In at 37 °C overnight. The RNA hybridized with antisense oligomers was subsequently enriched using Myone streptavidin C1 beads (65002; Invitrogen). After five-time washing with 37 °C prewarmed 2X SSC Wash buffer (0.5% SDS added), the enriched RNA was eluted with Proteinase K Buffer (100 mM NaCl, 10 mM Tris-Cl pH 7.0, 1 mM EDTA, 0.5% SDS) at 50 °C for 45 min. The eluted RNA from three pulldowns were then pooled together and purified using Trizol reagent, followed by cDNA synthesis and qPCR. The biotinylated antisense oligomers are listed in Supplementary Table 6.

## Supplementary information

Supplementary Material

Supplementary Table 1

Supplementary Table 2

Supplementary Table 3

Supplementary Table 4

Supplementary Table 5

Supplementary Table 6

## Data Availability

The datasets generated and/or analyzed during the current study are available from the corresponding author on reasonable request.

## References

[1] Piccolo S, Dupont S, Cordenonsi M (2014). The biology of YAP/TAZ: hippo signaling and beyond. Physiol Rev..

[2] Pan D (2010). The hippo signaling pathway in development and cancer. Dev Cell.

[3] Lei QY, Zhang H, Zhao B, Zha ZY, Bai F, Pei XH (2008). TAZ promotes cell proliferation and epithelial-mesenchymal transition and is inhibited by the hippo pathway. Mol Cell Biol..

[4] Zhao B, Li L, Tumaneng K, Wang CY, Guan KL (2010). A coordinated phosphorylation by Lats and CK1 regulates YAP stability through SCF(beta-TRCP). Genes Dev..

[5] Liu CY, Zha ZY, Zhou X, Zhang H, Huang W, Zhao D (2010). The hippo tumor pathway promotes TAZ degradation by phosphorylating a phosphodegron and recruiting the SCF{beta}-TrCP E3 ligase. J Biol Chem..

[6] Zhao B, Ye X, Yu J, Li L, Li W, Li S (2008). TEAD mediates YAP-dependent gene induction and growth control. Genes Dev..

[7] Zhang H, Liu CY, Zha ZY, Zhao B, Yao J, Zhao S (2009). TEAD transcription factors mediate the function of TAZ in cell growth and epithelial-mesenchymal transition. J Biol Chem..

[8] Anbanandam A, Albarado DC, Nguyen CT, Halder G, Gao X, Veeraraghavan S (2006). Insights into transcription enhancer factor 1 (TEF-1) activity from the solution structure of the TEA domain. Proc Natl Acad Sci USA.

[9] Plouffe SW, Lin KC, Moore JL, Tan FE, Ma S, Ye Z (2018). The Hippo pathway effector proteins YAP and TAZ have both distinct and overlapping functions in the cell. J Biol Chem..

[10] Dong J, Feldmann G, Huang J, Wu S, Zhang N, Comerford SA (2007). Elucidation of a universal size-control mechanism in *Drosophila* and mammals. Cell..

[11] Xie D, Yin D, Tong X, O’Kelly J, Mori A, Miller C (2004). Cyr61 is overexpressed in gliomas and involved in integrin-linked kinase-mediated Akt and beta-catenin-TCF/Lef signaling pathways. Cancer Res.

[12] Babic AM, Kireeva ML, Kolesnikova TV, Lau LF (1998). CYR61, a product of a growth factor-inducible immediate early gene, promotes angiogenesis and tumor growth. Proc Natl Acad Sci USA.

[13] Sun ZJ, Wang Y, Cai Z, Chen PP, Tong XJ, Xie D (2008). Involvement of Cyr61 in growth, migration, and metastasis of prostate cancer cells. Br J Cancer.

[14] Moya IM, Halder G (2019). Hippo-YAP/TAZ signalling in organ regeneration and regenerative medicine. Nat Rev Mol Cell Biol..

[15] Zanconato F, Cordenonsi M, Piccolo S (2016). YAP/TAZ at the roots of cancer. Cancer Cell.

[16] Van Roosbroeck K, Calin GA (2017). Cancer Hallmarks and microRNAs: the therapeutic connection. Adv Cancer Res.

[17] Schmitt AM, Chang HY (2016). Long noncoding RNAs in cancer pathways. Cancer Cell.

[18] Zhu B, Mitheera V, Finch-Edmondson M, Lee Y, Wan Y, Sudol M (2021). miR-582-5p is a tumor suppressor microRNA targeting the Hippo-YAP/TAZ signaling pathway in non-small cell lung cancer. Cancers.

[19] Zhang ZW, Men T, Feng RC, Li YC, Zhou D, Teng CB (2013). miR-375 inhibits proliferation of mouse pancreatic progenitor cells by targeting YAP1. Cell Physiol Biochem.

[20] Higashi T, Hayashi H, Ishimoto T, Takeyama H, Kaida T, Arima K (2015). miR-9-3p plays a tumour-suppressor role by targeting TAZ (WWTR1) in hepatocellular carcinoma cells. Br J Cancer.

[21] Shen S, Guo X, Yan H, Lu Y, Ji X, Li L (2015). A miR-130a-YAP positive feedback loop promotes organ size and tumorigenesis. Cell Res.

[22] Zhang Y, Huang W, Ran Y, Xiong Y, Zhong Z, Fan X (2015). miR-582-5p inhibits proliferation of hepatocellular carcinoma by targeting CDK1 and AKT3. Tumour Biol..

[23] Kang W, Huang T, Zhou Y, Zhang J, Lung RWM, Tong JHM (2018). miR-375 is involved in Hippo pathway by targeting YAP1/TEAD4-CTGF axis in gastric carcinogenesis. Cell Death Dis..

[24] Tan BS, Yang MC, Singh S, Chou YC, Chen HY, Wang MY (2019). LncRNA NORAD is repressed by the YAP pathway and suppresses lung and breast cancer metastasis by sequestering S100P. Oncogene..

[25] Li C, Wang S, Xing Z, Lin A, Liang K, Song J (2017). A ROR1-HER3-lncRNA signalling axis modulates the Hippo-YAP pathway to regulate bone metastasis. Nat Cell Biol..

[26] Lee Y, Finch-Edmondson M, Cognart H, Zhu B, Song H, Chuan LB (2020). Common and unique transcription signatures of YAP and TAZ in gastric cancer cells. Cancers.

[27] Tarazona S, Garcia-Alcalde F, Dopazo J, Ferrer A, Conesa A (2011). Differential expression in RNA-seq: a matter of depth. Genome Res.

[28] Tarazona S, Furio-Tari P, Turra D, Pietro AD, Nueda MJ, Ferrer A (2015). Data quality aware analysis of differential expression in RNA-seq with NOISeq R/Bioc package. Nucleic Acids Res.

[29] Hong W, Guan KL (2012). The YAP and TAZ transcription co-activators: key downstream effectors of the mammalian Hippo pathway. Semin Cell Dev Biol..

[30] Yagi R, Chen LF, Shigesada K, Murakami Y, Ito YA (1999). WW domain-containing yes-associated protein (YAP) is a novel transcriptional co-activator. EMBO J..

[31] Kanai F, Marignani PA, Sarbassova D, Yagi R, Hall RA, Donowitz M (2000). TAZ: a novel transcriptional co-activator regulated by interactions with 14-3-3 and PDZ domain proteins. EMBO J..

[32] Carithers LJ, Ardlie K, Barcus M, Branton PA, Britton A, Buia SA (2015). A novel approach to high-quality postmortem tissue procurement: the GTEx project. Biopreserv Biobank.

[33] Pobbati AV, Hong W (2013). Emerging roles of TEAD transcription factors and its coactivators in cancers. Cancer Biol Ther..

[34] Vassilev A, Kaneko KJ, Shu H, Zhao Y, DePamphilis ML (2001). TEAD/TEF transcription factors utilize the activation domain of YAP65, a Src/Yes-associated protein localized in the cytoplasm. Genes Dev..

[35] Zanconato F, Forcato M, Battilana G, Azzolin L, Quaranta E, Bodega B (2015). Genome-wide association between YAP/TAZ/TEAD and AP-1 at enhancers drives oncogenic growth. Nat Cell Biol..

[36] Chen LL (2016). Linking long noncoding RNA localization and function. Trends Biochem Sci..

[37] Pessa HK, Will CL, Meng X, Schneider C, Watkins NJ, Perala N (2008). Minor spliceosome components are predominantly localized in the nucleus. Proc Natl Acad Sci USA.

[38] Natalizio BJ, Wente SR (2013). Postage for the messenger: designating routes for nuclear mRNA export. Trends Cell Biol..

[39] Li F, Ambrosini G, Chu EY, Plescia J, Tognin S, Marchisio PC (1998). Control of apoptosis and mitotic spindle checkpoint by survivin. Nature.

[40] Shin S, Sung BJ, Cho YS, Kim HJ, Ha NC, Hwang JI (2001). An anti-apoptotic protein human survivin is a direct inhibitor of caspase-3 and -7. Biochemistry.

[41] Kim W, Cho YS, Wang X, Park O, Ma X, Kim H (2019). Hippo signaling is intrinsically regulated during cell cycle progression by APC/C(Cdh1). Proc Natl Acad Sci. USA.

[42] Qiao Y, Chen J, Lim YB, Finch-Edmondson ML, Seshachalam VP, Qin L (2017). YAP Regulates Actin Dynamics through ARHGAP29 and Promotes Metastasis. Cell Rep..

[43] Zhao B, Li L, Wang L, Wang CY, Yu J, Guan KL (2012). Cell detachment activates the Hippo pathway via cytoskeleton reorganization to induce anoikis. Genes Dev..

[44] Gumbiner BM, Kim NG (2014). The Hippo-YAP signaling pathway and contact inhibition of growth. J Cell Sci..

[45] Zhao B, Li L, Lu Q, Wang LH, Liu CY, Lei Q (2011). Angiomotin is a novel Hippo pathway component that inhibits YAP oncoprotein. Genes Dev..

[46] Zhou Z, Hao Y, Liu N, Raptis L, Tsao MS, Yang X (2011). TAZ is a novel oncogene in non-small cell lung cancer. Oncogene..

[47] Campbell JD, Alexandrov A, Kim J, Wala J, Berger AH, Pedamallu CS (2016). Distinct patterns of somatic genome alterations in lung adenocarcinomas and squamous cell carcinomas. Nat Genet.

[48] Graber TE, Hebert-Seropian S, Khoutorsky A, David A, Yewdell JW, Lacaille JC (2013). Reactivation of stalled polyribosomes in synaptic plasticity. Proc Natl Acad Sci USA.

[49] Panda AC, Martindale JL, Gorospe M (2017). Polysome fractionation to analyze mRNA distribution profiles. Bio Protoc.

[50] Lu Z, Zhang QC, Lee B, Flynn RA, Smith MA, Robinson JT (2016). RNA duplex map in living cells reveals higher-order transcriptome structure. Cell.

[51] Aw JG, Shen Y, Wilm A, Sun M, Lim XN, Boon KL (2016). In vivo mapping of eukaryotic RNA interactomes reveals principles of higher-order organization and regulation. Mol Cell.

[52] Garrett-Wheeler E, Lockard RE, Kumar A (1984). Mapping of psoralen cross-linked nucleotides in RNA. Nucleic Acids Res.

[53] Hearst JE (1981). Psoralen photochemistry and nucleic acid structure. J Invest Dermatol.

[54] Kim T, Yang SJ, Hwang D, Song J, Kim M, Kyum Kim S (2015). A basal-like breast cancer-specific role for SRF-IL6 in YAP-induced cancer stemness. Nat. Commun..

[55] Chang YC, Wu JW, Hsieh YC, Huang TH, Liao ZM, Huang YS (2018). Rap1 negatively regulates the Hippo pathway to polarize directional protrusions in collective cell migration. Cell Rep..

[56] Moroishi T, Park HW, Qin B, Chen Q, Meng Z, Plouffe SW (2015). A YAP/TAZ-induced feedback mechanism regulates Hippo pathway homeostasis. Genes Dev..

[57] Seavey CN, Pobbati AV, Hallett A, Ma S, Reynolds JP, Kanai R (2021). WWTR1(TAZ)-CAMTA1 gene fusion is sufficient to dysregulate YAP/TAZ signaling and drive epithelioid hemangioendothelioma tumorigenesis. Genes Dev..

[58] Driskill JH, Zheng Y, Wu BK, Wang L, Cai J, Rakheja D (2021). WWTR1(TAZ)-CAMTA1 reprograms endothelial cells to drive epithelioid hemangioendothelioma. Genes Dev..

[59] Strnadel J, Choi S, Fujimura K, Wang H, Zhang W, Wyse M (2017). eIF5A-PEAK1 signaling regulates YAP1/TAZ protein expression and pancreatic cancer cell growth. Cancer Res.

[60] Mayr C (2017). Regulation by 3’-untranslated regions. Annu Rev Genet.

[61] Mazumder B, Seshadri V, Fox PL (2003). Translational control by the 3’-UTR: the ends specify the means. Trends Biochem Sci..

[62] Tay Y, Kats L, Salmena L, Weiss D, Tan SM, Ala U (2011). Coding-independent regulation of the tumor suppressor PTEN by competing endogenous mRNAs. Cell..

[63] Cesana M, Cacchiarelli D, Legnini I, Santini T, Sthandier O, Chinappi M (2011). A long noncoding RNA controls muscle differentiation by functioning as a competing endogenous RNA. Cell.

[64] He RZ, Luo DX, Mo YY (2019). Emerging roles of lncRNAs in the post-transcriptional regulation in cancer. Genes Dis..

[65] Sarbassov DD, Guertin DA, Ali SM, Sabatini DM (2005). Phosphorylation and regulation of Akt/PKB by the rictor-mTOR complex. Science.

[66] Franken NA, Rodermond HM, Stap J, Haveman J, van Bree C (2006). Clonogenic assay of cells in vitro. Nat Protoc..

[67] Euhus DM, Hudd C, LaRegina MC, Johnson FE (1986). Tumor measurement in the nude mouse. J Surg Oncol..

[68] Tomayko MM, Reynolds CP (1989). Determination of subcutaneous tumor size in athymic (nude) mice. Cancer Chemother. Pharm..

[69] Martin M (2011). Cutadapt removes adapter sequences from high-throughput sequencing reads. EMBnet. journal.

[70] Dobin A, Davis CA, Schlesinger F, Drenkow J, Zaleski C, Jha S (2013). STAR: ultrafast universal RNA-seq aligner. Bioinformatics..

[71] Liao Y, Smyth GK, Shi W (2014). featureCounts: an efficient general purpose program for assigning sequence reads to genomic features. Bioinformatics..

[72] Love MI, Huber W, Anders S (2014). Moderated estimation of fold change and dispersion for RNA-seq data with DESeq2. Genome Biol..

[73] Kuleshov MV, Diaz JEL, Flamholz ZN, Keenan AB, Lachmann A, Wojciechowicz ML (2019). modEnrichr: a suite of gene set enrichment analysis tools for model organisms. Nucleic Acids Res.

