# Supplementary material for: LncRNA SFTA1P mediates positive feedback regulation of the Hippo-YAP/TAZ signaling pathway in non-small cell lung cancer
Source: Cell Death Discov. 2021 Nov 29;7:369. doi: 10.1038/s41420-021-00761-0 (PMC8630011; doi:10.1038/s41420-021-00761-0)
Supplement: Supplementary file 1 — Supplementary Material [file 41420_2021_761_MOESM1_ESM.pdf]

Supplementary Materials

Supplementary Figures

Figure S1. SFTA1P is a lung specific lncRNA transcriptionally regulated by YAP/TAZ/TEAD

Figure S2. Loss of SFTA1P inhibits cell proliferation and induces cell apoptosis in NSCLC cells

Figure S3. Knockdown of SFTA1P reduces YAP/TAZ transcriptional targets and TAZ protein in NSCLC cells

Supplementary Tables

Supplementary Table 1 MKN28_RNA_seq_lncRNA analysis

Supplementary Table 2 H1299_RNA_seq_DEGs analysis

Supplementary Table 3 Oligos for plasmid construction

Supplementary Table 4 siRNA used in the study

Supplementary Table 5 qPCR primers utilized in the study

Supplementary Table 6 Biotin-probes for RNA pulldown

Figure S1

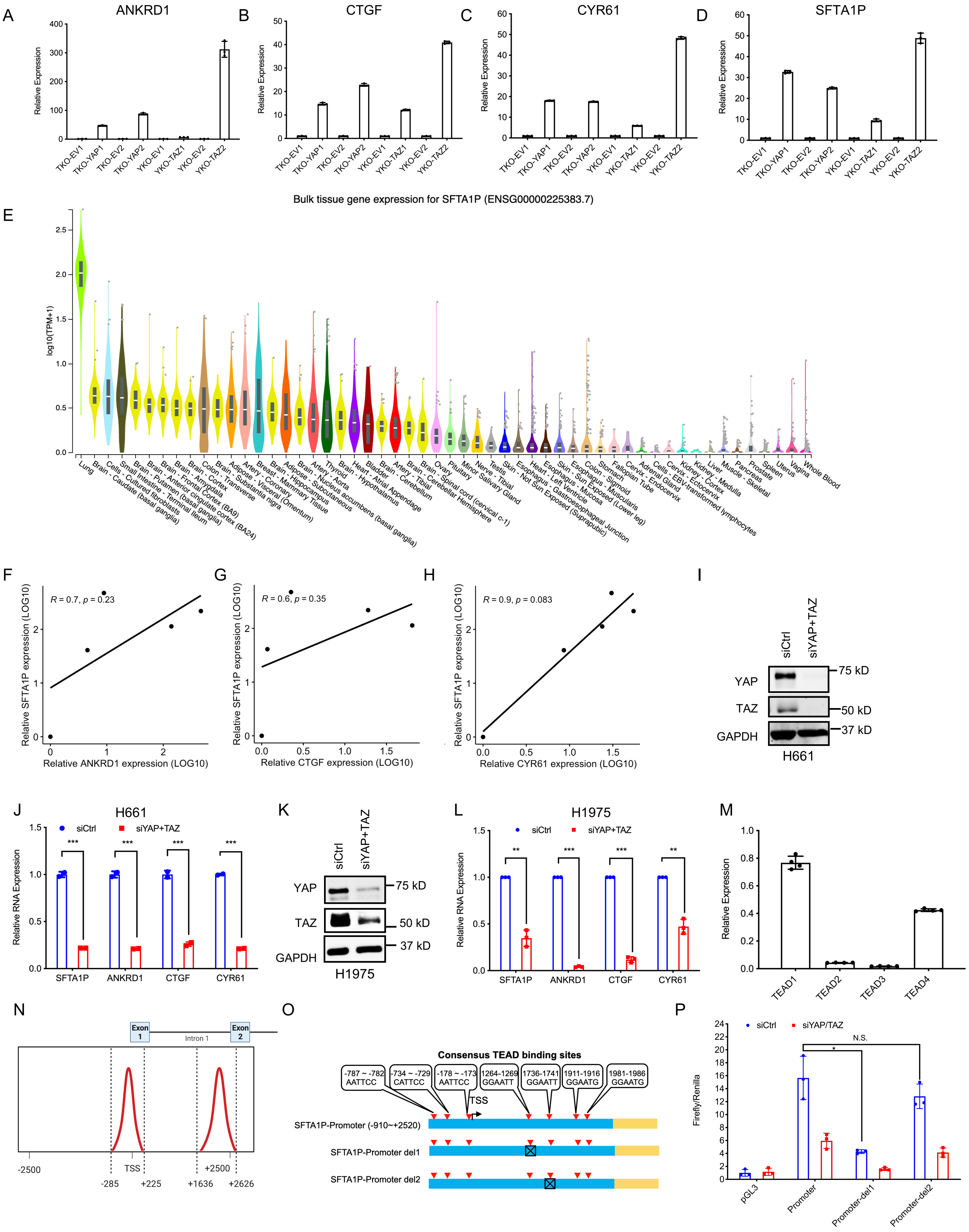

A
ANKRD1
B
CTGF
C
CYR61
D
SFTA1P
Relative Expression
TKO-EV1
TKO-YAP1
TKO-EV2
TKO-YAP2
YKO-EV1
YKO-TAZ1
YKO-EV2
YKO-TAZ2
Bulk tissue gene expression for SFTA1P (ENSG00000225383.7)
E
log10(TPM+1)
F
G
H
R = 0.7, p = 0.23
R = 0.6, p = 0.35
R = 0.9, p = 0.083
Relative SFTA1P expression (LOG10)
Relative ANKRD1 expression (LOG10)
Relative CTGF expression (LOG10)
Relative CYR61 expression (LOG10)
I
siCtrl
siYAP+TAZ
YAP
TAZ
GAPDH
75 kD
50 kD
37 kD
H661
J
H661
siCtrl
siYAP+TAZ
Relative RNA Expression
SFTA1P
ANKRD1
CTGF
CYR61
K
H1975
L
H1975
M
TEAD1
TEAD2
TEAD3
TEAD4
N
Exon 1
Intron 1
Exon 2
TSS
-2500
+2500
-285
+225
+1636
+2626
O
Consensus TEAD binding sites
-787 ~ -782 AATTCC
-734 ~ -729 CATTCC
-178 ~ -173 AATTCC
1264-1269 GGAATT
1736-1741 GGAATT
1911-1916 GGAATG
1981-1986 GGAATG
SFTA1P-Promoter (-910~+2520)
SFTA1P-Promoter del1
SFTA1P-Promoter del2
P
siCtrl
siYAP/TAZ
N.S.
Firefly/Renilla
pGL3
Promoter
Promoter-del1
Promoter-del2

Figure S1. SFTA1P is a lung specific lncRNA transcriptionally regulated by YAP/TAZ/TEAD.

(A-D) RT-qPCR analysis of ANKRD1(A), CTGF(B), CYR61 (C), and SFTA1P (D) in the engineered MKN28 utilized in the RNA-seq analysis. Mean ± S.D., n=3 (biological replicates) (E) The expression of SFTA1P in human tissues. The data were obtained from GTEx Analysis Release V7 (dbGaP Accession phs000424.v7.p2). (F-H) Correlations between the expression of SFTA1P and transcriptional targets of YAP/TAZ, namely ANKRD1 (F), CTGF (G), and CYR61 (H). The correlation coefficient (*R-value*) and the significance (*p-value*) were obtained by performing Spearman correlation analysis. Each dot represents the mean expression of 3 biological replicates (I-L) Knockdowns of YAP and TAZ in H661 and H1975 cell lines. Western blot analysis of YAP and TAZ following the treatments of siRNAs in H661 (I) and H1975 (K) cell lines. RT-qPCR analysis of SFTA1P, ANKRD1, CTGF, and CYR61 in H661 (J) and H1975 (L) cell lines post-treated with indicated siRNAs. (M) RT-qPCR analysis of four transcripts encoded by the TEAD genes belonging to the TEAD family in H1299 cells. Relative expression of TEAD1/2/3/4 is calculated by normalizing the ΔCT of each TEAD against that of 18S rRNA. Mean ± S.D., n= 4 (biological replicates). (N) Published ChIP-seq data reveal two putative YAP/TAZ/TEADs binding regions proximal to the TSS of SFTA1P. (O) A schematic illustration of the sequence spanning from -910 bp to +2 520 bp neighboring the SFTA1P's TSS (Blue). The red triangles pinpoint the consensus TEAD binding sequences (5'-CATTCCA/T-3' or 5'-GGAATT/G-3'). The wild type sequence (SFTA1P-Promoter) and two deletion mutants (SFTA1P-Promoter del1/2) were each cloned into the 5' end of a firefly luciferase gene (yellow) in the pGL3 plasmid. (P) Luciferase reporter assay was performed to examine the relative activity of empty vector (pGL3), or the indicated recombinant vectors upon siRNA knockdown of YAP/TAZ. Mean ± S.D. n=3 (biological replicates). Statistical analysis was conducted using Student's t-test, *$p<0.05$, **$p<0.01$, ***$p<0.001$.

Figure S2

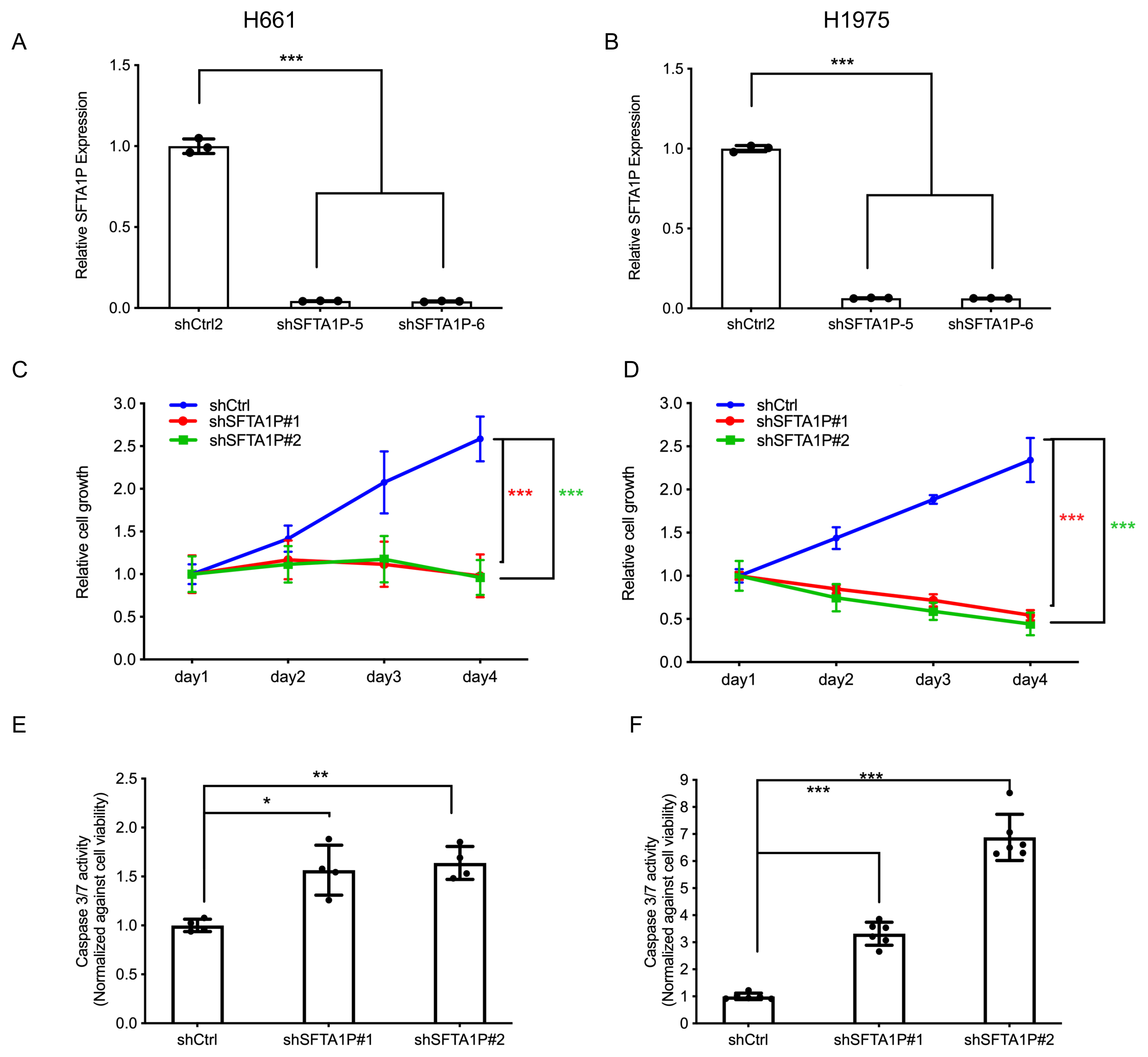

H661
H1975
A
B
C
D
E
F
Relative SFTA1P Expression
shCtrl2
shSFTA1P-5
shSFTA1P-6
***
Relative cell growth
shCtrl
shSFTA1P#1
shSFTA1P#2
day1
day2
day3
day4
Caspase 3/7 activity
(Normalized against cell viability)
*
**

Figure S2. Loss of SFTA1P inhibits cell proliferation and induces cell apoptosis in NSCLC cells.

(A-B) RT-qPCR analysis of SFTA1P expression in H661 (A) and H1975 (B) cells upon shRNA knockdown. Mean ± S.D., n=3 (biological replicates). (C-D) The cell growth curve was measured by cell viability assay following the knockdown of SFTA1P in H661 (C) and H1975 (D) cell lines. Mean ± S.D. n=4 or 6 (biological replicates). (E-F) Cell apoptosis was measured using Caspase 3/7 assay in H661 (E) and H1975 (F) cell lines. Mean ± S.D. n=4 or 6 (biological replicates). Student's t-test was used in statistical analysis, *p<0.05, **p<0.01, ***p<0.001.

Figure S3

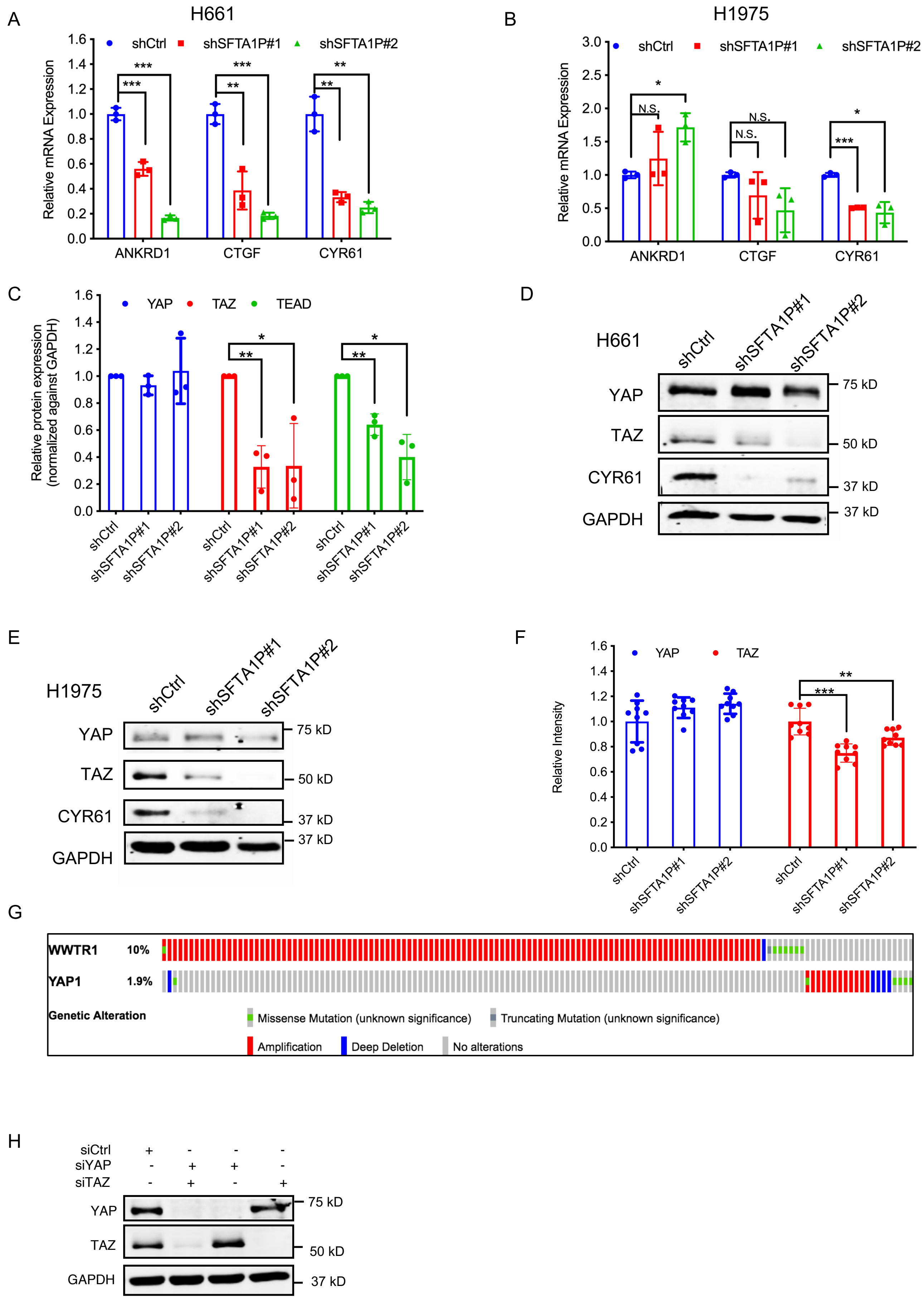

A
H661
shCtrl
shSFTA1P#1
shSFTA1P#2
Relative mRNA Expression
1.6
1.4
1.2
1.0
0.8
0.6
0.4
0.2
0.0
***
***
***
**
**
**
ANKRD1
CTGF
CYR61
B
H1975
shCtrl
shSFTA1P#1
shSFTA1P#2
Relative mRNA Expression
3.0
2.5
2.0
1.5
1.0
0.5
0.0
*
N.S.
N.S.
N.S.
*
***
ANKRD1
CTGF
CYR61
C
YAP
TAZ
TEAD
Relative protein expression
(normalized against GAPDH)
1.6
1.4
1.2
1.0
0.8
0.6
0.4
0.2
0.0
*
**
*
**
shCtrl
shSFTA1P#1
shSFTA1P#2
shCtrl
shSFTA1P#1
shSFTA1P#2
shCtrl
shSFTA1P#1
shSFTA1P#2
D
H661
shCtrl
shSFTA1P#1
shSFTA1P#2
YAP
TAZ
CYR61
GAPDH
75 kD
50 kD
37 kD
37 kD
E
H1975
shCtrl
shSFTA1P#1
shSFTA1P#2
YAP
TAZ
CYR61
GAPDH
75 kD
50 kD
37 kD
37 kD
F
YAP
TAZ
Relative Intensity
1.6
1.4
1.2
1.0
0.8
0.6
0.4
0.2
0.0
**
***
shCtrl
shSFTA1P#1
shSFTA1P#2
shCtrl
shSFTA1P#1
shSFTA1P#2
G
WWTR1 10%
YAP1 1.9%
Genetic Alteration
Missense Mutation (unknown significance)
Truncating Mutation (unknown significance)
Amplification
Deep Deletion
No alterations
H
siCtrl + - - -
siYAP - + + -
siTAZ - + - +
YAP
TAZ
GAPDH
75 kD
50 kD
37 kD

Figure S3. Knockdown of SFTA1P reduces YAP/TAZ transcriptional targets and TAZ protein in NSCLC cells.

(A-B) RT-qPCR analysis of YAP/TAZ transcriptional targets was performed in H661 (A) and H1975 (B) cells following the shRNA-mediated SFTA1P knockdowns. Mean ± S.D., n=3 (biological replicates). (C) Related to Figure 3C. Western blot quantification of YAP, TAZ, and TEADs protein expression in shRNA-treated H1299 cells. Mean ± S.D., n=3 (biological replicates). (D-E) western blot analysis of YAP, TAZ, and CYR61 in H661 (D) and H1975 (E) cells following shRNA treatments. (F) Related to Figure 3F. A quantitative analysis of immunofluorescence staining results showing the relative intensity of YAP and TAZ. Mean ± S.D., n=9 (biological replicates). (G) Frequencies of the genetic alterations (e.g., missense mutation, truncating mutation, amplification, and deletion) of YAP (YAP1) and TAZ (WWTR1) in NSCLC clinical samples. Data were derived from c-bioportal. (H) Western blot analysis of YAP and TAZ upon siRNA treatment in H1299 cells. Statistical significance was determined using Student's t-test, *p<0.05, **p<0.01, ***p<0.001.
