# Supplementary material for: LncRNA SFTA1P mediates positive feedback regulation of the Hippo-YAP/TAZ signaling pathway in non-small cell lung cancer
Source: Cell Death Discov. 2021 Nov 29;7:369. doi: 10.1038/s41420-021-00761-0 (PMC8630011; doi:10.1038/s41420-021-00761-0)
Supplement: Supplementary file 4 — Supplementary Table 3 [file 41420_2021_761_MOESM4_ESM.docx]

Supplementary Table 3. Oligos for plasmid construction

| Oligos | Sequence |
| --- | --- |
| shCtrl-F | 5’-CCGGTCCTAAGGTTAAGTCGCCCTCGCTCGAGCGAGGGCGACTTAACCTTAGGTTTTTG-3’ |
| shCtrl-R | 5’-AATTCAAAAACCTAAGGTTAAGTCGCCCTCGCTCGAGCGAGGGCGACTTAACCTTAGGA-3’ |
| shSFTA1P#1-F | 5'-CCGGGGATGGTCAGAGAGGAGATTACTCGAGTAATCTCCTCTCTGACCATCCTTTTTG-3' |
| shSFTA1P#1-R | 5'-AATTCAAAAAGGATGGTCAGAGAGGAGATTACTCGAGTAATCTCCTCTCTGACCATCC-3' |
| shSFTA1P#2-F | 5'-CCGGCCAGCTCCACCACTCAATAAACTCGAGTTTATTGAGTGGTGGAGCTGGTTTTTG-3' |
| shSFTA1P#2-R | 5'-AATTCAAAAACCAGCTCCACCACTCAATAAACTCGAGTTTATTGAGTGGTGGAGCTGG-3' |
| SFTA1P-promoter-F | 5’-CAGCCACGCGTGGCAAATGCAGGGAATAACTAAAA T-3’ |
| SFTA1P-promoter-R  SFTA1P-promoter-del1-F  SFTA1P-promoter-del1-R  SFTA1P-promoter-del2-F  SFTA1P-promoter-del2-F | 5’-CAGCCAGATCTGCAATATTCCGGGTTAGGAATTCCATC -3’  ﻿5’-CCATACGGTCTTCTCGTC-3’  ﻿5’-CTAATTTTCCATATTTGAAGAAATAGG-3’  ﻿5’GCTATTATGGGACATTTG-3’  5’-TAAGTCATCGATGAAGGAC-3’ |
| TAZ-3’-UTR-1F | 5’-CAGCGCGGCCGCTCACTACCATTGTAACTTGGATGTAGC-3’ |
| TAZ-3’-UTR-1R | 5’-CAGCGTCGACAGATTCTAAGCTGCAATTTTTTAAATCC-3’ |
| TAZ-3’-UTR-2F | 5’-CAGCGCGGCCGCCAAAAGTTTTTGAAATATTACAACTGG-3’ |
| TAZ-3’-UTR-2R | 5’-CAGCGTCGACCAGTTGAGGACTTCATTGGCAATG-3’ |
| TAZ-3’-UTR-3F | 5’-CAGCGCGGCCGCCAACTGGCATGCAGTCTGCCTG-3’ |
| TAZ-3’-UTR-3R | 5’-CAGCGTCGACGCCACAAGCTTACTAGAAAATTACTTC-3’ |
| TAZ-3’-UTR-4F | 5’-CAGCGCGGCCGCGTAAATCATTGATGATTTATATTACCAATTTTTAG-3’ |
| TAZ-3’-UTR-4R | 5’-CAGCGTCGACCTGTTTCCTGTATATGGTGTAATCAG-3’ |
| TAZ-3’-UTR-5F | 5’-CAGCGCGGCCGCGATGTGAAATGCCATTTCTTTCACTG-3’ |
| TAZ-3’-UTR-5R | 5’-CAGCGTCGACTGGAAGTTCAATTGTCTTTATTTTTCTTATACAG-3’ |
| hTAZ-FLAG-F-BamHI | 5’-CGTGGATCCGCCACCATGGACTACAAAGACGATGACGACAAGAATCCGGCCTCGGCGCCCCCTC-3’ |
| hTAZ-R-MluI&XhoI | 5’-CGTCTCGAGACGCGTTTACAGCCAGGTTAGAAAGG-3’ |
