# Supplementary material for: LncRNA SFTA1P mediates positive feedback regulation of the Hippo-YAP/TAZ signaling pathway in non-small cell lung cancer
Source: Cell Death Discov. 2021 Nov 29;7:369. doi: 10.1038/s41420-021-00761-0 (PMC8630011; doi:10.1038/s41420-021-00761-0)
Supplement: Supplementary file 5 — Supplementary Table 4 [file 41420_2021_761_MOESM5_ESM.docx]

Supplementary Table 4. siRNA used in the study

| siRNA | Identifiers |
| --- | --- |
| siCtrl | Cat.4390846 |
| siYAP | Cat 4392420 (s20366; s20367; s20368) |
| siTAZ | Cat 4392420 (s24787; s24788; s24789) |
| siTEAD1 | Cat 4392420 (s13961; s13962; s13963) |
| siTEAD4 | Cat 4392420 (s13964; s13965; s13966) |
