# Supplementary material for: LncRNA SFTA1P mediates positive feedback regulation of the Hippo-YAP/TAZ signaling pathway in non-small cell lung cancer
Source: Cell Death Discov. 2021 Nov 29;7:369. doi: 10.1038/s41420-021-00761-0 (PMC8630011; doi:10.1038/s41420-021-00761-0)
Supplement: Supplementary file 6 — Supplementary Table 5 [file 41420_2021_761_MOESM6_ESM.docx]

Supplementary Table 5. qPCR primers utilized in the study

| Gene | Forward | Reverse |
| --- | --- | --- |
| TEAD 1 | 5’-GGCCGGGAATGATTCAAACAG-3’ | 5’-CAATGGAGCGACCTTGCCA-3’ |
| TEAD 2 | 5’-GCCTCCGAGAGCTATATGATCG-3’ | 5’-TCACTCCGTAGAAGCCACCA-3’ |
| TEAD 3 | 5’-TCATCCTGTCAGACGAGGG-3’ | 5’-TCTTCCGAGCTAGAACCTGTATG-3’ |
| TEAD 4 | 5’-GGACACTACTCTTACCGCATCC-3’ | 5’-TCAAAGACATAGGCAATGCACA-3’ |
| YAP | 5’-TAGCCCTGCGTAGCCAGTTA-3’ | 5’-TCATGCTTAGTCCACTGTCTGT-3’ |
| TAZ | 5’-GGACCTAGACACAGACCTCG-3’ | 5’-GCCCGAATCAGGCTCCTTAA-3’ |
| ANKRD1 | 5’-CGAGATAAGTTGCTCAGCACAG-3’ | 5’-GTTCAGTCTCACCGCATCATG-3’ |
| CTGF | 5’-TGCCCTCGCGGCTTACCGACTG-3’ | 5’-TGCAGGAGGCGTTGTCATTGGTAAC-3’ |
| CYR61 | 5’-GGTCAAAGTTACCGGGCAGT-3’ | 5’-GGAGGCATCGAATCCCAGC-3’ |
| SFTA1P | 5’-TCTCTACTTCCCTGTGACCTC-3’ | 5’-TGATCTTTCTCCGCAGTGTG-3’ |
| GAPDH | 5’-AAGGGCATCCTGGGCTACACTGAG-3’ | 5’-GAAATGAGCTTGACAAAGTTGTCGTT-3’ |
| 18SrRNA | 5’-GTAACCCGTTGAACCCCATT-3’ | 5’-CCATCCAATCGGTAGTAGCG-3’ |
| U2 | 5’-TGGAGCAGGGAGATGGAATA-3’ | 5’-CGTTCCTGGAGGTACTGCAA-3’ |
| beta-actin | 5’-TCCCTGGAGAAGAGCTACGA-3’ | 5’-AGCACTGTGTTGGCGTACAG-3’ |
