# Supplementary material for: LncRNA SFTA1P mediates positive feedback regulation of the Hippo-YAP/TAZ signaling pathway in non-small cell lung cancer
Source: Cell Death Discov. 2021 Nov 29;7:369. doi: 10.1038/s41420-021-00761-0 (PMC8630011; doi:10.1038/s41420-021-00761-0)
Supplement: Supplementary file 7 — Supplementary Table 6 [file 41420_2021_761_MOESM7_ESM.docx]

Supplementary Table 6. Biotin-probes for RNA pulldown

| Probes | Sequence |
| --- | --- |
| 1 | 5’-ACCTGGAATGCTGTATAACC-3’BioTEG |
| 2 | 5’-TAAGATGAGCTTCCACGGAT-3’BioTEG |
| 3 | 5’-CGGCTTTCCTTGAAAACTCA-3’BioTEG |
| 4 | 5’-AGCAGGTGTTGACATTGTTT-3’BioTEG |
| 5 | 5’-CTCGTTGTAATTCAAGGGCA-3’BioTEG |
| 6 | 5’-CATCTCACTTGAAGAGGTGC-3’BioTEG |
| 7 | 5’-AATACATTTCCATCCTGAGG-3’BioTEG |
| 8 | 5’-GGAAGTAGAGAGGGGGATGA-3’BioTEG |
| 9 | 5’-TCCATTCTTCTGTTCTTCTG-3’BioTEG |
| 10 | 5’-GTCTTGTCAATGTTCAGTCA-3’BioTEG |
| 11 | 5’-TAATCTCCTCTCTGACCATC-3’BioTEG |
| 12 | 5’-AAATGATCTTTCTCCGCAGT-3’BioTEG |
| 13 | 5’-TTGGTGAGATGGATGGGGAG-3’BioTEG |
| 14 | 5’-GTTTTATTGAGTGGTGGAGC-3’BioTEG |
